# Molecular Insights into Bromocriptine Binding to GPCRs Within Histamine-Linked Signaling Networks: Network Pharmacology, Pharmacophore Modeling, and Molecular Dynamics Simulation

**DOI:** 10.3390/ijms26178717

**Published:** 2025-09-07

**Authors:** Doni Dermawan, Lamiae Elbouamri, Samir Chtita, Nasser Alotaiq

**Affiliations:** 1 Department of Applied Biotechnology, Faculty of Chemistry, Warsaw University of Technology, 00-661 Warsaw, Poland; doni.dermawan.stud@pw.edu.pl; 2 Laboratory of Analytical and Molecular Chemistry, Faculty of Sciences Ben M’Sik, Hassan II University of Casablanca, Casablanca 20670, Morocco; elbouamrilamiae14@gmail.com (L.E.); samirchtita@gmail.com (S.C.); 3 Health Sciences Research Center (HSRC), Imam Mohammad Ibn Saud Islamic University (IMSIU), Riyadh 13317, Saudi Arabia

**Keywords:** bromocriptine, histamine, molecular dynamics, network pharmacology, pharmacophore modeling

## Abstract

This study aimed to investigate the molecular binding mechanisms of bromocriptine toward histamine-associated targets, exploring both antagonist-like and other potential interaction modes that may support therapeutic repurposing. Network pharmacology was applied to identify histamine-related pathways and prioritize potential protein targets. CXCR4, GHSR, and OXTR were selected based on combined docking scores and pharmacophore modeling evidence. Molecular dynamics (MD) simulations over 100 ns assessed structural stability, flexibility, compactness, and solvent exposure. Binding site contact analysis and MM/PBSA free binding energy calculations were conducted to characterize binding energetics and interaction persistence. Bromocriptine exhibited stable binding to all three receptors, engaging key residues implicated in receptor modulation (e.g., Asp187 in CXCR4, Asp99 in GHSR, Arg232 in OXTR). The MM/PBSA ΔG_binding values of bromocriptine were −22.67 ± 3.70 kcal/mol (CXCR4 complex), −22.11 ± 3.55 kcal/mol (GHSR complex), and −21.43 ± 2.41 kcal/mol (OXTR complex), stronger than standard agonists and comparable to antagonists. Contact profiles revealed shared and unique binding patterns across targets, reflecting their potential for diverse modulatory effects. Bromocriptine demonstrates high-affinity binding to multiple histamine-associated GPCR targets, potentially exerting both inhibitory and modulatory actions. These findings provide a molecular basis for further experimental validation and therapeutic exploration in histamine-related conditions.

## 1. Introduction

Histamine is a biogenic amine that mediates various physiological and pathological processes via its four canonical G protein-coupled receptors: histamine receptor H1 (H_1_R), H2 (H_2_R), H3 (H_3_R), and H4 (H_4_R). Each receptor subtype has distinct tissue distributions, ligand affinities, and signaling pathways contributing to diverse roles in inflammation, neurotransmission, and gastric physiology [1,2,3]. H_1_R is predominantly involved in allergic responses and neuroinflammation, while H_2_R regulates gastric acid secretion in the gastrointestinal tract [4]. In contrast, H_3_R and H_4_R are high-affinity receptors with specialized roles in the central nervous and immune systems. H_3_R, primarily expressed in the brain, acts as a presynaptic auto-receptor that modulates the release of neurotransmitters, including histamine, dopamine, acetylcholine, and serotonin [5,6]. H_4_R, expressed on hematopoietic cells and microglia, participates in chemotaxis, cytokine release, and immune surveillance [7].

Dysregulation of histaminergic signaling has been implicated in multiple disease states, including allergic conditions, neurodegenerative diseases, and inflammatory disorders [8,9]. Notably, growing evidence has highlighted the importance of histamine-dopamine crosstalk, particularly in the pathophysiology of Parkinson’s disease and related neurodegenerative conditions [10]. For instance, excessive histaminergic activity via H_1_R is known to contribute to microglial activation and oxidative stress in the substantia nigra, exacerbating dopaminergic neuron degeneration [11,12]. H_3_R has been shown to modulate dopamine and γ-aminobutyric acid (GABA) levels in basal ganglia circuits, which are crucial for motor control and cognition [13]. Additionally, H_4_R plays a role in neuroimmune signaling, with reported effects on blood–brain barrier integrity and neuroinflammation [9,14]. These findings have positioned histamine receptors as attractive therapeutic targets in neurodegenerative and neuroinflammatory diseases.

Bromocriptine is a semi-synthetic ergot alkaloid and a potent dopamine D_2_ receptor agonist. Clinically, it is employed in treating hyperprolactinemia, Parkinson’s disease, and type 2 diabetes mellitus [15,16]. The therapeutic effects of bromocriptine stem from its high-affinity binding and functional agonism at D_2_ receptors, though it also exhibits interactions with serotonergic (5-HT_1_A, 5-HT_2_B) and adrenergic receptors [17]. Beyond its classical dopaminergic effects, recent studies have uncovered anti-inflammatory properties of bromocriptine. It has been shown to inhibit nitric oxide production and cytokine release in RAW264.7 macrophages and reduce mast cell degranulation in RBL-2H3 cells [18]. Intriguingly, bromocriptine has also demonstrated the ability to attenuate histamine-stimulated gastric acid secretion in experimental models [19,20]. Despite these findings, bromocriptine’s direct molecular interaction with histamine receptors or histamine-related biological pathways has not yet been systematically studied. The potential intersection of bromocriptine’s pharmacological profile with histaminergic systems opens new opportunities for drug repurposing, especially in diseases where both dopamine and histamine signaling are dysregulated, such as Parkinson’s disease, schizophrenia, and multiple sclerosis. A systems-level exploration of bromocriptine’s interactions with histamine-associated targets may reveal novel polypharmacological mechanisms and therapeutic implications.

Advances in computational drug discovery now enable the integration of network pharmacology with structure-based simulation techniques to characterize drug–target interactions on a systems scale [21,22,23]. Network pharmacology allows for identifying intersecting targets and pathways shared between drugs and diseases, providing insight into pleiotropic effects and off-target interactions [24]. Structural modeling approaches, such as pharmacophore modeling, molecular docking, and molecular dynamics (MD) simulations, can validate and refine the predicted interactions, offering atomistic resolution into ligand–receptor binding events [25,26]. Despite increasing interest in histamine receptor modulators, no prior computational study has comprehensively assessed bromocriptine’s potential interaction with histamine-related targets.

In this study, we aimed to comprehensively investigate the potential interactions between bromocriptine and histamine-associated targets using a multiscale computational approach. We first identified the intersection between bromocriptine-related and histamine-related target proteins through integrated network pharmacology analysis, leveraging publicly available databases such as GeneCards, NCBI Gene, and OMIM. Protein–protein interaction (PPI) networks and pathway enrichment analyses were conducted to elucidate the biological significance of these overlapping targets. Pharmacophore modeling was performed to identify shared chemical features between bromocriptine and known histamine receptor ligands. Molecular docking and MD simulations were applied to validate specific target engagements and assess binding affinity, interaction stability, and key residue contacts. Finally, MM/PBSA calculations were used to estimate the binding free energy of bromocriptine at selected receptor sites. Through this integrative approach, we aimed to elucidate the polypharmacological landscape of bromocriptine in relation to histamine signaling, providing novel insights into its mechanism of action and potential repositioning opportunities in neuroinflammatory and histamine-mediated disorders.

## 2. Results

### 2.1. Network Pharmacology-Based Target Mapping Reveals Histamine-Related Polypharmacology of Bromocriptine

**Figure 1:**
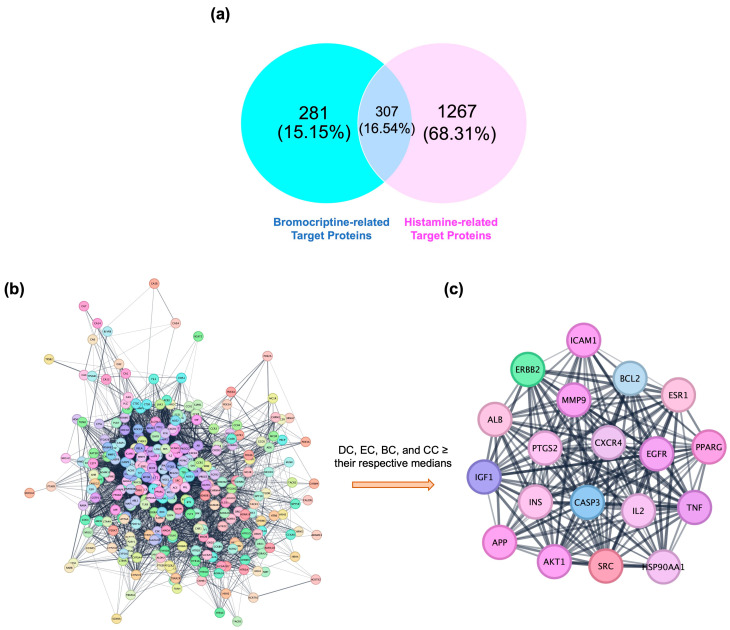
Network-based identification of bromocriptine–histamine-associated core targets. (**a**) Venn diagram showing the overlap between predicted bromocriptine-related targets (blue) and histamine-associated targets curated from GeneCards, NCBI Gene, and OMIM (pink). A total of 307 proteins were found in common, representing putative histamine-related targets of bromocriptine. (**b**) Protein–protein interaction (PPI) network constructed from the 307 overlapping proteins using the STRING database. The network comprises 302 nodes and 5477 edges, demonstrating a densely interconnected architecture with extensive functional cross-talk. (**c**) Hub subnetwork extracted from the PPI network in (**b**) by applying topological filtering based on four centrality metrics (degree centrality ≥ 17.0, eigenvector centrality ≥ 0.220, betweenness centrality ≥ 0.118, closeness centrality ≥ 0.947). This analysis reduced the global network to 19 highly interconnected hub proteins forming a tightly clustered module. The dense reciprocal connections suggest these hubs act as critical control points coordinating bromocriptine’s potential modulation of histamine-related signaling pathways.

An integrative target-mapping approach, combining cheminformatics predictions with disease-associated gene repositories, was employed to investigate potential histamine-related mechanisms of bromocriptine. Comparative analysis revealed a notable intersection of 307 shared targets between bromocriptine and histamine-associated protein sets (Figure 1a). This overlap accounted for 16.54% of the unique proteins identified, with bromocriptine-exclusive targets representing 15.15% and histamine-exclusive targets representing 68.31%. The substantial intersection suggests that bromocriptine, traditionally recognized for dopaminergic receptor agonism, may also modulate histamine-related pathways, indicating a broader pharmacological profile. This finding supports the hypothesis of bromocriptine’s potential polypharmacological action in disorders where dopamine–histamine interactions are pathophysiologically significant. The Venn diagram in Figure 1a represents the first-level overlap analysis between predicted bromocriptine targets (obtained from SEA, DrugBank, STITCH, TargetNet, and PharmMapper) and curated histamine-associated proteins (obtained from GeneCards, NCBI Gene, and OMIM). The shared 307 proteins were then extracted and subjected to STRING-based protein–protein interaction (PPI) analysis to assess their network-level interconnectivity (Figure 1b). This explains the transition from the overlap (Figure 1a) to the full PPI network (Figure 1b). Functional relationships among the intersecting targets were explored by constructing a PPI network. The network comprised 302 nodes and 5477 edges (Figure 1b), reflecting a dense interconnectivity indicative of functional cooperation and co-regulation within histamine-related signaling systems. The high edge-to-node ratio suggests that perturbation of even a single highly connected node could trigger cascading effects across multiple pathways. The absence of isolated clusters further indicates a unified network in which modulation of one target could propagate through extensive biological processes.

Subsequently, to derive Figure 1c from Figure 1b, we applied a stepwise topological filtering approach. Specifically, four centrality parameters (degree centrality (DC), eigenvector centrality (EC), betweenness centrality (BC), and closeness centrality (CC)) were calculated for every node in the PPI network. Nodes exceeding predefined thresholds (DC ≥ 17.00, EC ≥ 0.220, BC ≥ 0.118, CC ≥ 0.947) were designated as hubs. This reduced the 302-node global PPI network to a compact core of 19 highly interconnected hub proteins, visualized as Figure 1c. Thus, Figure 1c is not an independent network, but rather a filtered subnetwork derived directly from Figure 1b by applying topological metrics. This analysis identified 19 core hub proteins (Figure 1c) forming a compact, highly interconnected subnetwork, with dense and reciprocal edges suggesting strong functional associations and possible co-regulation. Biological characterization of these 19 hubs revealed multiple proteins implicated in neuroinflammation and neurodegeneration, including TNF (tumor necrosis factor), IL2 (interleukin-2), ICAM1 (intercellular adhesion molecule 1), and CXCR4 (C-X-C chemokine receptor type 4), all central to immune cell trafficking, cytokine signaling, and inflammatory cascades [27,28,29]. Apoptosis-related regulators such as CASP3 (caspase-3) and BCL2 suggest potential involvement in neuronal survival pathways, relevant to dopaminergic neuron preservation in neurodegenerative disorders [30,31]. Growth factor and kinase signaling proteins, including EGFR (epidermal growth factor receptor), IGF1 (insulin-like growth factor 1), AKT1 (AKT serine/threonine kinase 1), and SRC (proto-oncogene tyrosine-protein kinase Src), indicate convergence with cell proliferation, differentiation, and synaptic plasticity mechanisms [32,33,34]. Additional targets such as APP (amyloid precursor protein) implicate potential connections to Alzheimer’s disease pathology, while MMP9 (matrix metalloproteinase-9) may modulate extracellular matrix remodeling and blood–brain barrier integrity [35,36]. The diversity of these hub proteins underscores the possibility that bromocriptine’s histamine-related target network spans multiple biological systems, including immune modulation, neuronal survival, vascular function, and extracellular matrix regulation. Such multifunctional engagement suggests that bromocriptine holds repurposing potential for a broad spectrum of histamine-mediated pathologies, ranging from neuroinflammatory diseases such as multiple sclerosis to neurodegenerative disorders including Parkinson’s and Alzheimer’s disease. The complete network pharmacology results can be seen in Supplementary Data S1.

**Figure 2:**
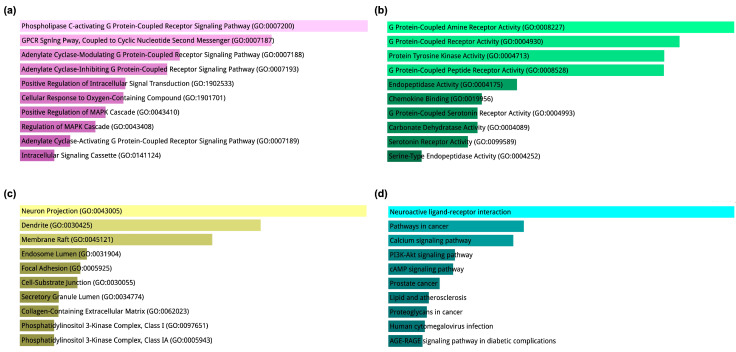
Functional enrichment analysis of the 19 core hub proteins/receptors. (**a**) Gene Ontology (GO) Biological Process (BP) enrichment showing significant association with G protein-coupled receptor (GPCR) signaling pathways, regulation of the MAPK cascade, and intracellular signal transduction. (**b**) GO Molecular Function (MF) enrichment dominated by GPCR activities, protein tyrosine kinase activity, chemokine binding, and neurotransmitter receptor activity. (**c**) GO Cellular Component (CC) enrichment indicating predominant localization to neuron projections, dendrites, membrane rafts, and collagen-containing extracellular matrix, as well as association with the phosphatidylinositol 3-kinase (PI3K) complex. (**d**) Kyoto Encyclopedia of Genes and Genomes (KEGG) pathway enrichment highlighting neuroactive ligand–receptor interaction, PI3K–Akt signaling, cAMP signaling, calcium signaling, and multiple disease-related pathways. Color coding: pink (BP), green (MF), yellow-olive (CC), and cyan-teal (KEGG).

The Gene Ontology (GO) enrichment analysis, based on the 19 core hub proteins/receptors, was categorized into Biological Process (BP), Molecular Function (MF), and Cellular Component (CC), along with Kyoto Encyclopedia of Genes and Genomes (KEGG) signaling pathways. This multi-dimensional analysis provides a systems-level view of the functional roles and signaling contexts in which these hub targets operate. In the BP category (Figure 2a), the most enriched terms were associated with G protein-coupled receptor (GPCR)-related signaling, including phospholipase C-activating GPCR signaling pathway (GO:0007200) and adenylate cyclase-modulating GPCR signaling pathway (GO:0007188). Both stimulatory and inhibitory adenylate cyclase GPCR pathways were represented, indicating a capacity for bidirectional regulation of cyclic nucleotide second messengers. Other highly enriched processes included positive regulation of intracellular signal transduction, MAPK cascade regulation, and cellular responses to oxygen-containing compounds, suggesting that these receptors are central to environmental signal detection, kinase cascade activation, and cellular stress adaptation. This aligns with the expected multi-functional nature of hub proteins like EGFR, IGF1, AKT1, and SRC, which bridge extracellular ligand binding with intracellular signaling responses.

The MF category (Figure 2b) reinforced these findings, significantly enriching G protein-coupled amine receptor activity, G protein-coupled receptor activity, and protein tyrosine kinase activity, representing both ligand-binding and catalytic functions. Enrichment in chemokine binding and serotonin receptor activity highlights the potential cross-talk between neurotransmission and immune modulation, which is highly relevant to neuroinflammation and neuronal repair contexts. Carbonate dehydratase activity and serine-type endopeptidase activity further indicate metabolic and proteolytic regulatory roles within the hub protein network. In the CC category (Figure 2c), the top hits, neuron projection, dendrite, and membrane raft point to a strong neuronal and synaptic localization, particularly within lipid-rich signaling microdomains that facilitate receptor clustering and intracellular signaling initiation. Additional enrichment in endosome lumen and secretory granule lumen suggests involvement in vesicular trafficking and regulated exocytosis. Structural terms such as collagen-containing extracellular matrix indicate a role in extracellular architecture remodeling, potentially linked to synaptic plasticity or repair. The enrichment of phosphatidylinositol 3-kinase (PI3K) complex components is consistent with activation of PI3K–Akt survival signaling by several of the hub proteins. KEGG pathway analysis (Figure 2d) placed neuroactive ligand–receptor interaction as the most significantly enriched pathway, highlighting the role of neurotransmitter and neuromodulator signaling in the system. Pathways such as PI3K–Akt signaling, cAMP signaling, and calcium signaling connect directly with the BP and MF findings, indicating convergent intracellular routes triggered by receptor activation. The enrichment of disease-related pathways, including pathways in cancer, lipid and atherosclerosis, AGE–RAGE signaling in diabetic complications, and human cytomegalovirus infection, suggests broader systemic relevance and potential links between neuronal receptor activity, metabolic regulation, and inflammatory pathophysiology. These results reveal that the 19 core hub proteins/receptors are positioned at the interface of extracellular ligand recognition, intracellular kinase cascade activation, and neuronal structural localization. Integrating GPCR, tyrosine kinase, and PI3K–Akt pathways underscores their potential as multifunctional regulators in neuroprotective, neuroinflammatory, and regenerative contexts.

**Figure 3:**
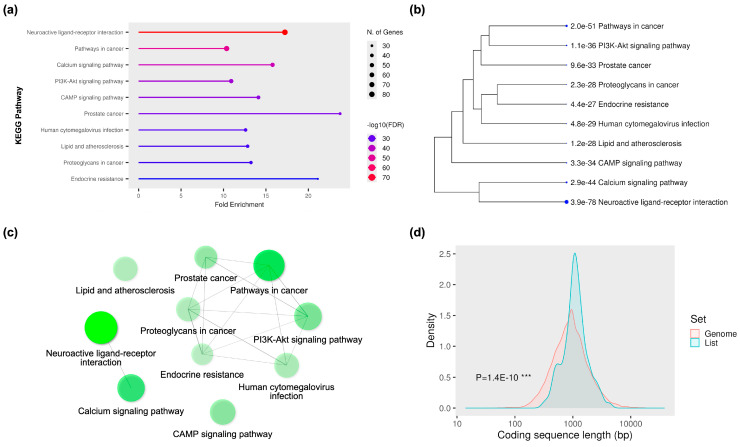
KEGG pathway enrichment and coding sequence analysis of the 19 core hub proteins/receptors. (**a**) KEGG pathway enrichment dot plot showing the top 10 significantly enriched pathways, with point size representing the number of genes and color intensity indicating –log10(FDR). The neuroactive ligand–receptor interaction pathway showed the highest enrichment, followed by calcium signaling, PI3K–Akt signaling, and cAMP signaling pathways, along with multiple disease-related pathways. (**b**) Hierarchical clustering dendrogram of KEGG pathways based on gene overlap, revealing functional grouping between related signaling and disease modules. (**c**) Pathway–pathway interaction network visualization, with node size proportional to enrichment significance, highlighting the central position of neuroactive ligand–receptor interaction in the pathway network. (**d**) Coding sequence (CDS) length distribution comparison between the enriched gene set (blue) and the reference genome (red), with statistical significance determined by the Kolmogorov–Smirnov test (*p* = 1.4 × 10^−10^). *** indicates a highly significant difference (*p* < 0.001).

The KEGG pathway enrichment analysis of the 19 core hub proteins revealed multiple signaling and disease-related pathways, many closely associated with neurodegeneration, neuroinflammation, and cancer-related signaling. In Figure 3a, the top enriched pathway was neuroactive ligand–receptor interaction, showing the highest fold enrichment and statistical significance. This was followed by signaling pathways such as calcium signaling, PI3K–Akt signaling, and cAMP signaling, which are crucial for neuronal excitability, synaptic plasticity, and neuroprotective mechanisms [37,38]. In addition to these neurobiological pathways, several cancer-related and metabolic pathways, including pathways in cancer, prostate cancer, lipid and atherosclerosis, and proteoglycans in cancer, were also enriched, reflecting the pleiotropic roles of the identified hubs in cell proliferation, immune modulation, and vascular integrity. The hierarchical clustering dendrogram in Figure 3b illustrates the relationships between these enriched pathways based on gene overlap. Closely related pathways, such as PI3K–Akt signaling, endocrine resistance, and proteoglycans in cancer, suggested functional convergence in growth factor signaling, cellular survival, and extracellular matrix interactions. Similarly, neuroactive ligand–receptor interaction and the calcium signaling pathway were closely linked, reflecting their shared roles in neurotransmitter-mediated signaling and calcium-dependent neuronal responses.

A network representation of the KEGG pathways (Figure 3c) further highlights the interconnectedness of these signaling modules. The neuroactive ligand–receptor interaction pathway emerged as a central hub in this network, linking with other signaling systems such as calcium signaling, PI3K–Akt, and cAMP. This network topology reinforces the idea that the 19 core hub proteins may function in a coordinated fashion to regulate neuronal and non-neuronal biological processes. Finally, Figure 3d shows a density distribution of coding sequence (CDS) lengths for genes in the enriched list compared to the reference genome. The observed CDS length distribution for the hub-associated genes significantly differed from the genome-wide distribution (*p* = 1.4 × 10^−10^), suggesting a non-random selection of genes with characteristic coding sequence properties. This may reflect evolutionary constraints or functional specializations of these hub proteins in maintaining signal transduction fidelity and structural complexity.

**Figure 4:**
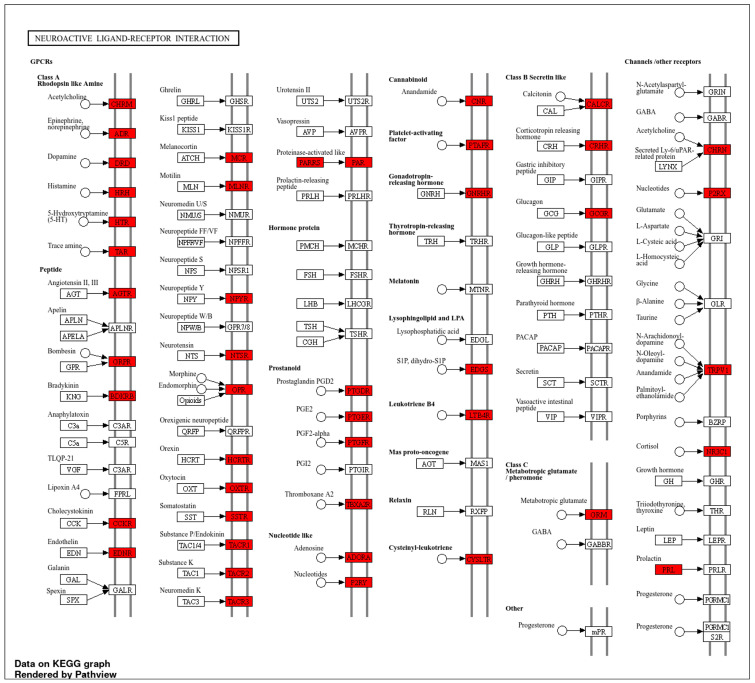
KEGG neuroactive ligand–receptor interaction pathway mapping of prioritized docking receptors. The diagram illustrates the distribution of the 32 selected receptor targets (highlighted in red) within the neuroactive ligand–receptor interaction pathway. The highlighted receptors represent the intersection of bromocriptine-related and histamine-related target proteins identified through network pharmacology screening. Pathway visualization was generated using Pathview based on KEGG database annotations.

Mapping the 19 core hub proteins to the KEGG neuroactive ligand–receptor interaction pathway revealed enrichment across diverse receptor families, including neurotransmitter, neuropeptide, hormone, cytokine, and ion channel receptors (Figure 4). From these, 32 receptors were prioritized for molecular docking based on functional relevance, tractable drug-binding properties, and their potential to interact with bromocriptine. Importantly, these 32 receptors are not redundant but occupy distinct KEGG pathway branches, each representing a unique docking context defined by ligand class, receptor family, and downstream signaling outcomes. For example, the histamine receptor family (HRH1, HRH2, HRH3, HRH4) is clustered within the aminergic GPCR branch, of particular interest given emerging evidence of histaminergic pathways in modulating neurotransmission, inflammatory signaling, and vascular tone [39,40]. This cluster highlights mechanistic domains where bromocriptine may exert modulatory effects, consistent with reports linking histamine receptor modulation to conditions such as migraine, sleep–wake regulation, and neuropsychiatric disorders [41]. This suggests that bromocriptine’s binding to these targets could extend its therapeutic profile beyond dopaminergic activity. The dopamine receptor family (DRD2, DRD3, DRD4) was also identified, with DRD2 included for redocking validation using its bromocriptine-bound crystal structure (PDB ID: 7JVR [42]), underscoring its well-established role as a primary bromocriptine target. Other functional clusters were also evident: serotonin receptors (HTR1A, HTR2A) within the aminergic GPCR group; chemokine signaling represented by CXCR4; neuropeptide signaling by GHSR, OXTR, NPY1R, NTSR1, and PRLR; nuclear hormone receptors including ESR1 and PPARG; adrenergic (ADRA1B) and cholinergic (CHRM1, CHRNA4) receptors; inflammatory mediators (C3AR1, PTGER3); glutamatergic (GRIN1, GRM3); and sensory transduction receptors (TRPV1, TAAR1). By explicitly mapping these distinct receptor families and pathway branches, Figure 4 demonstrates how bromocriptine may engage multiple signaling systems in a pathway-specific manner. This integrative mapping provides a strategic foundation for probing bromocriptine’s multi-receptor pharmacology, particularly its unexplored potential in histaminergic signaling. The complete chosen receptor database can be seen in Supplementary Data S2.

### 2.2. Binding Affinity and Interaction Profiles of Bromocriptine with Target Receptors Identified from Network Pharmacology

Molecular docking analysis revealed that bromocriptine exhibited favorable binding affinities toward multiple target receptors at the intersection of bromocriptine and histamine-associated proteins. The table presented here focuses on the top 10 bromocriptine–receptor complexes, ranked by HADDOCK score, with docking scores ranging from −62.9 to −40.9 a.u., corresponding to estimated binding free energies between −10.67 and −9.02 kcal/mol (Table 1). The growth hormone secretagogue receptor (GHSR) complex showed the most favorable HADDOCK score (−62.9 ± 1.6 a.u.), followed closely by CXCR4 (−56.1 ± 0.7 a.u.) and DRD2 (−57.4 ± 0.5 a.u.). Interestingly, CXCR4 exhibited the strongest predicted binding energy (−10.67 kcal/mol). In comparison, GHSR demonstrated a slightly lower binding energy (−10.36 kcal/mol) but achieved the best overall docking score, suggesting an optimal combination of van der Waals, electrostatic, and desolvation contributions. The complete molecular docking results for all chosen receptors are provided in Supplementary Data S3. Component-wise energy decomposition revealed distinct interaction profiles across complexes. Electrostatic interactions dominated in CXCR4 (−124.0 ± 9.1 kcal/mol) and HRH3 (−84.0 ± 3.6 kcal/mol), whereas van der Waals interactions were more prominent in DRD3 (−30.3 ± 0.3 kcal/mol) and OXTR (−32.0 ± 0.4 kcal/mol). The most favorable desolvation energy was observed for OXTR (−22.8 ± 1.3 kcal/mol), suggesting advantageous hydrophobic contributions to binding. RMSD values for all top-ranked complexes were ≤0.6 Å, reflecting the stability of the docking poses. A re-docking validation was performed using the bromocriptine-bound DRD2 crystal structure as a reference to ensure docking reliability. The re-docking reproduced the experimental binding pose with an RMSD of 0.2 ± 0.2 Å, confirming the accuracy and robustness of the docking protocol.

**Figure 5:**
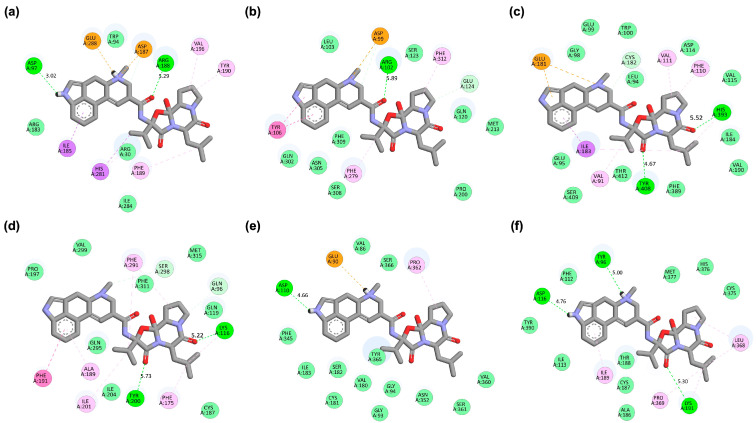
Two-dimensional interaction maps of top-performing bromocriptine-receptor complexes. (**a**) CXCR4_Bromocriptine complex. (**b**) GHSR_Bromocriptine complex. (**c**) DRD2_Bromocriptine complex. (**d**) OXTR_Bromocriptine complex. (**e**) DRD3_Bromocriptine complex. (**f**) HTR1A_Bromocriptine complex. The interaction types are color-coded as follows: hydrogen bonds (bright green), van der Waals interactions (pale green), Pi-Alkyl (pink), Pi-Sigma (purple), Pi-Sulfur (orange), and Halogen interactions (bright blue).

The 2D interaction maps (Figure 5) illustrate residue-specific contacts and their corresponding pharmacophoric features, thereby linking atomic binding patterns to functional group recognition and enabling comparison with agonist- and antagonist-derived pharmacophore models. In the CXCR4 complex (Figure 5a), bromocriptine forms hydrogen bond donor and acceptor interactions with Asp97 and Asp187, complemented by aromatic π–π stacking with Tyr190. This configuration mirrors antagonist-like pharmacophore features, including polar stabilization and aromatic anchoring, consistent with later pharmacophore modeling results (Supplementary Data S6). In GHSR (Figure 5b), hydrogen bonding with Arg102 is accompanied by hydrophobic/aromatic pharmacophore contacts with Tyr106 and Phe312. These hydrophobic anchor points overlap with those engaged by the antagonist JMV-2959, unlike the agonist GHRP-6, which displays a more limited hydrophobic profile. The DRD2 complex (Figure 5c) shows hydrogen bonding with His393 and Tyr408 alongside van der Waals/aromatic pharmacophore interactions with Phe389 and Thr412, consistent with its canonical dopaminergic binding mode. For OXTR (Figure 5d), hydrogen bonding with Lys116 and Tyr200 appears critical to ligand stabilization, mapping onto basic donor–acceptor pharmacophore elements characteristic of the antagonist atosiban. DRD3 (Figure 5e) and HTR1A (Figure 5f) exhibit a mixed pharmacophore profile combining H-bonding, hydrophobic contacts, and aromatic stacking, indicating partial overlap with known agonist and antagonist binding motifs. Thus, while the residue-level maps highlight specific atomic contacts, the pharmacophore-level interpretation underscores recurring motifs, hydrogen-bond donors/acceptors, hydrophobic/aromatic groups, and charged interactions that position bromocriptine closer to antagonist-like profiles in CXCR4, GHSR, and OXTR, while maintaining canonical dopaminergic engagement.

The molecular interaction profile analysis (Table 2) revealed that carbon–carbon (CC) interactions were the most dominant across all bromocriptine–receptor complexes, with the GHSR_Bromocriptine complex exhibiting the highest CC count (3658), followed by DRD2_Bromocriptine (3298) and CXCR4_Bromocriptine (3179). Carbon–oxygen (CO) and carbon–nitrogen (CN) interactions were also prevalent, generally ranging between 892 and 1545, and 1091 and 1414 interactions, respectively. Notably, GHSR_Bromocriptine and CXCR4_Bromocriptine consistently demonstrated higher CO and CN interaction counts compared to other complexes, suggesting stronger polar contact contributions. Carbon–any heavy atom (CX) interactions, although relatively low in frequency, were most prominent in DRD2_Bromocriptine (58) and HTR1A_Bromocriptine (62). Oxygen–oxygen (OO) and oxygen–any heavy atom (OX) interactions occurred at minimal levels, with the highest OO count in GHSR_Bromocriptine (162) and OX count in DRD2_Bromocriptine (10). Nitrogen–oxygen (NO) interactions were moderately frequent, with GHSR_Bromocriptine again showing the highest value (283), followed closely by DRD2_Bromocriptine (270). Nitrogen–nitrogen (NN) and nitrogen–any heavy atom (NX) interactions were comparatively rare, with the highest NN count (142) in EGFR_Bromocriptine and the highest NX count (14) in HTR1A_Bromocriptine. Interestingly, no any heavy–heavy atom (XX) interactions were detected in any complex, indicating the absence of direct heavy atom–heavy atom contacts without carbon, nitrogen, or oxygen mediation. These findings suggest that bromocriptine’s binding to various receptors is primarily driven by carbon-mediated hydrophobic contacts, supplemented by polar interactions involving oxygen and nitrogen atoms, with interaction patterns varying subtly between receptor types. The complete results of molecular interactions can be seen in Supplementary Data S4.

**Figure 6:**
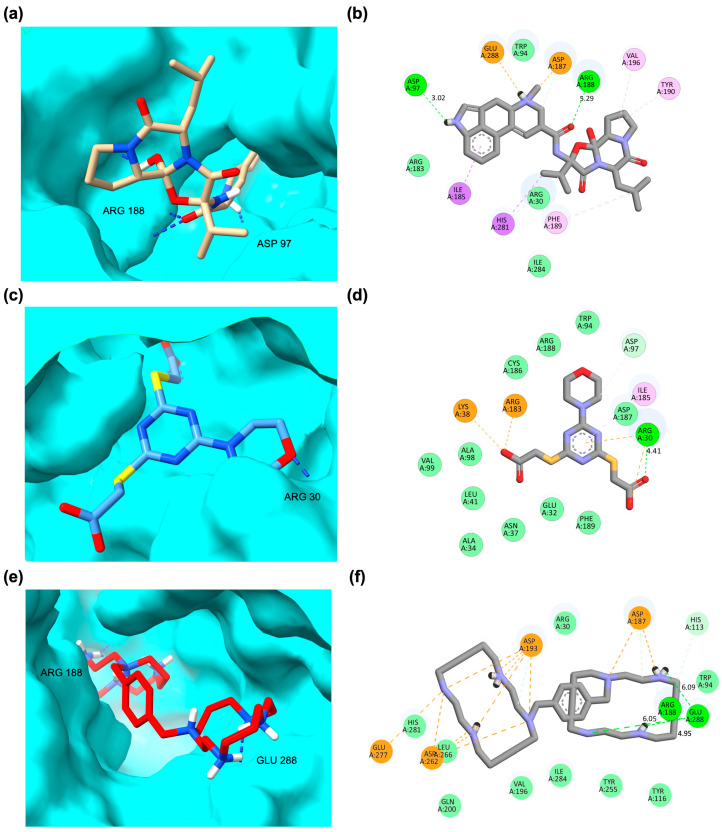
Comparative 3D and 2D binding pose of bromocriptine, standard agonists, and antagonists in the CXCR4 ligand-binding domain (LBD). (**a**) 3D binding pose of bromocriptine in CXCR4. (**b**) 2D interaction map of bromocriptine–CXCR4 complex showing hydrogen bonds with Asp97 and Arg188. (**c**) 3D binding pose of CXCL-12 (agonist) in CXCR4. (**d**) 2D interaction map of CXCL-12–CXCR4 complex highlighting hydrogen bonds and hydrophobic contacts. (**e**) 3D binding pose of Plerixafor (antagonist) in CXCR4. (**f**) 2D interaction map of Plerixafor–CXCR4 complex showing hydrogen bonding with Arg188 and Glu288. The interaction types are color-coded as follows: hydrogen bonds (bright green), van der Waals interactions (pale green), Pi-Alkyl (pink), Pi-Sigma (purple), Pi-Sulfur (orange), and Halogen interactions (bright blue). In the 3D binding poses, atoms are color-coded by element: oxygen (red), nitrogen (blue), and sulfur (yellow).

To address binding pocket flexibility, we employed a semi-flexible docking protocol where bromocriptine was fully flexible while key side chains in the receptor LBDs were allowed limited conformational adjustment during docking. Furthermore, all docking poses were subsequently validated through MD simulations, which explicitly account for full receptor and ligand flexibility in a solvated environment. This two-step strategy ensured that the proposed docking modes in Figure 6 are not rigid artifacts but rather conformationally stable poses consistent with dynamic pocket fluctuations. CXCR4, GHSR, and OXTR were prioritized for detailed molecular docking and MD simulations because their potential interactions with bromocriptine have not been previously explored, and preliminary screening indicated promising binding affinities. Interestingly, bromocriptine displayed notable similarities with specific standard ligands, including a shared hydrogen bond with Arg188 in the CXCR4 ligand-binding domain (LBD), a critical interaction also observed with the standard antagonist Plerixafor. Molecular docking analysis comparing bromocriptine with known agonists and antagonists revealed notable interaction similarities and differences across the CXCR4, GHSR, and OXTR LBDs (Table 3, Figure 6). For CXCR4, bromocriptine achieved a HADDOCK score of −56.1 ± 0.7 a.u. and a binding free energy of −10.67 kcal/mol, positioning it between the agonist CXCL-12 (−7.06 kcal/mol) and the potent antagonist Plerixafor (−14.21 kcal/mol). In addition to its interaction with Arg188, bromocriptine formed a hydrogen bond with Asp97 and engaged in aromatic stacking with Tyr190, contributing to electrostatic stabilization.

In GHSR, bromocriptine exhibited a strong docking performance with a HADDOCK score of −62.9 ± 1.6 a.u. and a binding energy of −10.36 kcal/mol, comparable to the antagonist JMV-2959 (−10.49 kcal/mol) and surpassing the agonist GHRP-6 (−8.58 kcal/mol). Key interactions included hydrogen bonds with Arg102 and hydrophobic contacts with Phe279 and Phe312, suggesting a potential modulatory role in receptor activity. For OXTR, bromocriptine showed favorable docking metrics (HADDOCK score −56.5 ± 0.9 a.u.; binding energy −9.93 kcal/mol), similar to the antagonist Atosiban and slightly lower than the agonist Oxytocin. Bromocriptine formed a key hydrogen bond with Lys116 in OXTR, the same residue targeted by Atosiban, along with another hydrogen bond to Tyr200 and favorable van der Waals interactions that help stabilize its binding. The 2D and 3D interactions of GHSR and OXTR complexes can be seen in Supplementary Data S5. Figures S1 and S2 show the comparative 3D and 2D binding pose of GHSR and OXTR complexes, respectively.

### 2.3. Pharmacophore Modeling Supports and Extends Molecular Docking Insights

**Figure 7:**
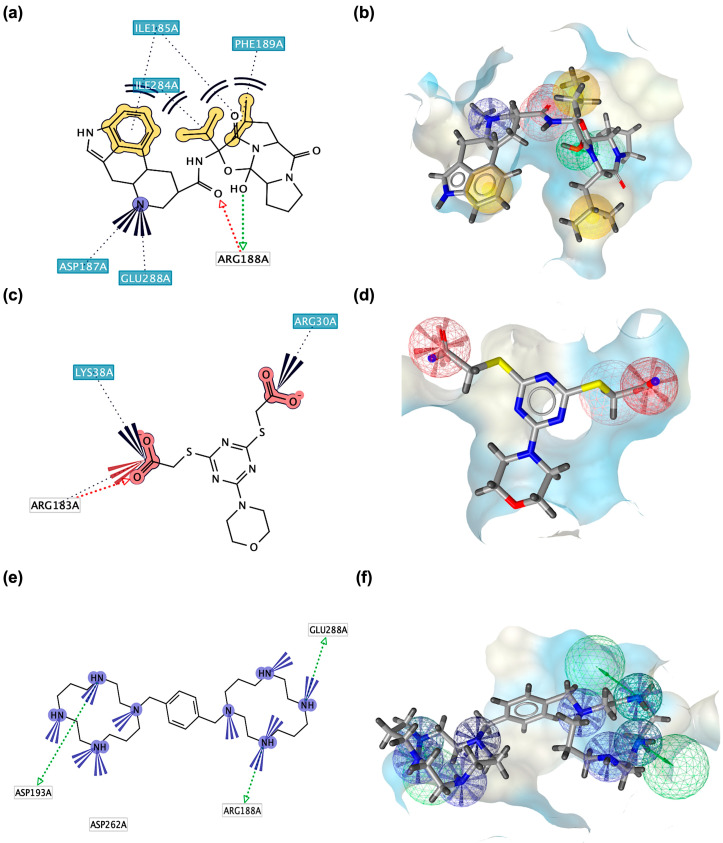
Pharmacophore modeling results of bromocriptine, standard agonists, and antagonists in the CXCR4 LBD. (**a**) 2D pharmacophore model of CXCR4_Bromocriptine. (**b**) 3D pharmacophore model of CXCR4_Bromocriptine. (**c**) 2D pharmacophore model of CXCR4_CXCL-12 (agonist). (**d**) 3D pharmacophore model of CXCR4_CXCL-12 (agonist). (**e**) 2D pharmacophore model of CXCR4_Plerixafor (antagonist). (**f**) 3D pharmacophore model of CXCR4_Plerixafor (antagonist). Yellow spheres indicate hydrophobic interactions, green arrows represent hydrogen bond donors, red arrows signify hydrogen bond acceptors, and blue star-shaped spheres represent positive ionizable groups.

Pharmacophore modeling further validated the molecular docking results for bromocriptine and reference ligands in the CXCR4 LBD (Figure 7). The pharmacophore model of the CXCR4_Bromocriptine complex (Figure 7a,b) revealed a combination of hydrogen bond donor and acceptor features, hydrophobic contacts, and a positive ionizable group interacting with Glu288, similar to the standard antagonist Plerixafor, closely mirroring key pharmacophoric elements seen in the standard antagonist Plerixafor. Bromocriptine exhibited hydrogen bond donor and acceptor interactions with Arg188, significantly stabilizing antagonist-like binding in CXCR4. Additional hydrophobic interactions with Ile185, Phe189, and Ile284 enhanced its binding complementarity within the LBD. In contrast, the pharmacophore profile of the CXCR4_CXCL-12 complex (Figure 7c,d) was dominated by hydrogen bond acceptor features at Arg30, Lys38, and Arg183, consistent with agonist-mediated activation patterns. This configuration differed markedly from bromocriptine’s binding, suggesting that while some hydrogen bonding overlap exists, bromocriptine’s pharmacophore aligns more closely with antagonist characteristics. The CXCR4_Plerixafor model (Figure 7e,f) demonstrated extensive hydrogen bond donor interactions, including Arg188, Asp193, and Glu288, and a dense network of positive ionizable sites. Bromocriptine shared the Arg188 hydrogen bond donor interaction, reinforcing the docking observation that it may partially mimic the binding strategy of this established antagonist. The pharmacophore mapping collectively underscores bromocriptine’s hybrid binding potential, retaining antagonist-like interactions such as Arg188 hydrogen bonding while engaging additional hydrophobic and polar contacts that could modulate receptor conformation.

The pharmacophore modeling of bromocriptine, standard agonists, and antagonists in the GHSR LBD revealed distinct interaction profiles. For bromocriptine, the 2D and 3D pharmacophore models showed multiple hydrophobic interactions with residues Val182, Phe279, Leu37, and Tyr106, along with hydrogen bond donor interactions involving Gln120 and acceptor interactions with Arg102. In the standard agonist GHRP-6, a dominant hydrophobic interaction was observed with Leu34, while for the antagonist JMV-2959, hydrophobic contacts with Tyr106, Leu103, and Phe286 were complemented by a positive ionizable group interaction near Glu124. Moving to OXTR complexes, bromocriptine exhibited extensive hydrophobic interactions with Ala189, Phe191, Ile201, Phe291, Ile204, and Phe175, as well as hydrogen bond acceptor contact with Tyr200 and hydrophobic proximity to Trp99. The agonist oxytocin showed hydrogen bond donor interactions with Ser298 and hydrophobic contact with Trp188. In contrast, the antagonist atosiban displayed a combination of hydrophobic interactions with Val299, Phe311, and Ala308, hydrogen bond acceptor contacts with Lys116, and a positive ionizable group near Lys306. These pharmacophore features indicate that bromocriptine retains multiple hydrophobic anchor points across both receptors but engages residue subsets that are more characteristic of antagonists. This inhibitory-leaning pharmacophoric fingerprint supports its classification alongside CXCR4, GHSR, and OXTR antagonists. It reinforces its selection for downstream MD simulations to explore receptor-specific binding stability and conformational effects. The pharmacophore modeling results of GHSR and OXTR complexes can be seen in Supplementary Data S6. Figures S3 and S4 show the pharmacophore profiles for GHSR and OXTR complexes, respectively.

### 2.4. MD Simulations Corroborate the Robustness and Binding Consistency of the Ligand–Receptor Complexes

**Figure 8:**
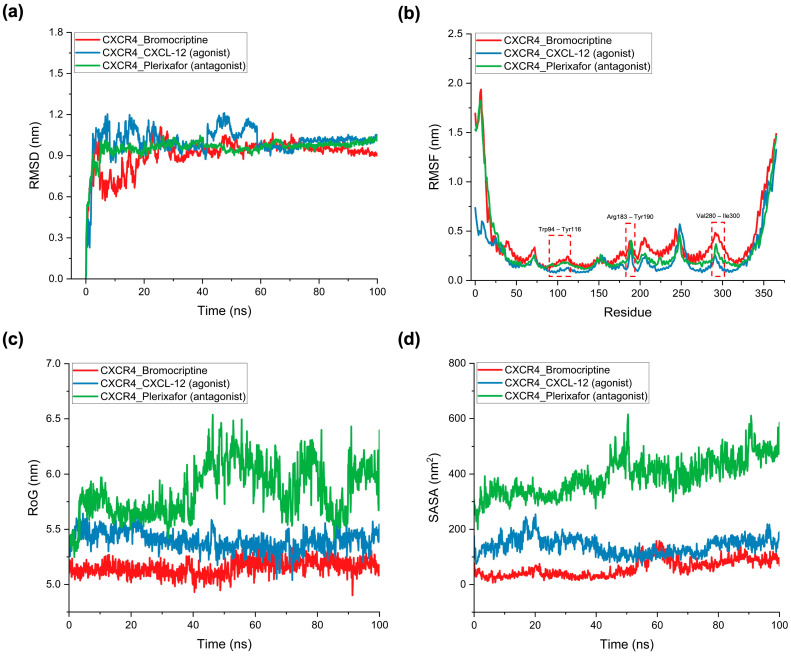
MD simulation profiles of bromocriptine, standard agonists, and antagonists bound to the CXCR4 LBD. (**a**) Root mean square deviation (RMSD). (**b**) Root mean square fluctuation (RMSF). (**c**) Radius of gyration (RoG). (**d**) Solvent-accessible surface area (SASA).

To further validate docking and pharmacophore results and gain insight into the dynamic behavior of bromocriptine in the CXCR4 LBD, comparative MD simulations were conducted alongside a standard agonist (CXCL-12) and antagonist (Plerixafor). Four key structural parameters, including root mean square deviation (RMSD), root mean square fluctuation (RMSF), radius of gyration (RoG), and solvent-accessible surface area (SASA), were monitored over 100 ns to assess global stability, residue-level flexibility, compactness, and solvent exposure of the complexes. RMSD analysis (Figure 8a) demonstrated that the bromocriptine–CXCR4 complex reached equilibrium within ~10 ns and maintained an average RMSD of ~0.9 nm for the remainder of the simulation. This stability profile closely paralleled that of the antagonist Plerixafor (~0.95 nm), whereas the agonist CXCL-12 exhibited higher RMSD values (~1.0–1.1 nm) with more pronounced oscillations throughout the trajectory. These observations suggest that bromocriptine adopts a persistent binding orientation within the LBD, comparable to the antagonist, and is less prone to large-scale conformational drift than the agonist complex. The minimal RMSD fluctuations in bromocriptine and Plerixafor indicate a well-packed binding conformation that resists destabilization under dynamic conditions. RMSF analysis (Figure 8b) revealed distinct flexibility profiles across the receptor. Three critical ligand-binding segments—Trp94–Tyr116, Arg183–Tyr190, and Val280–Ile300—displayed nearly identical fluctuation amplitudes in the bromocriptine and Plerixafor complexes, whereas CXCL-12-bound CXCR4 exhibited noticeably dampened fluctuations in these same regions. The elevated mobility in bromocriptine and Plerixafor suggests partial destabilization of local hydrogen bonding networks between the ligand and these residues, which may hinder the conformational shifts necessary for receptor activation. This disruption of stabilizing interactions is a hallmark of antagonistic binding behavior. Conversely, the reduced flexibility in the agonist complex indicates reinforced contacts that promote an activation-competent conformation of CXCR4.

RoG profiles (Figure 8c) further differentiated agonist and antagonist binding modes. Bromocriptine maintained a compact global conformation (~5.4 nm) comparable to Plerixafor (~5.5 nm), with minimal fluctuations throughout the simulation, while CXCL-12 displayed a steady increase in RoG values (~5.5 to >6.5 nm). This gradual expansion in the agonist complex likely reflects activation-associated conformational changes that increase the overall molecular dimensions of the receptor–ligand system. Maintaining a smaller, more rigid structural envelope in bromocriptine supports its functional resemblance to antagonist binding. SASA measurements (Figure 8d) also aligned with this interpretation. Bromocriptine and Plerixafor consistently exhibited low solvent exposure (~300–400 nm^2^) across the simulation window, indicating that the ligand–receptor interfaces remained tightly packed and shielded from solvent penetration. In contrast, the agonist complex showed a progressive increase in SASA, surpassing 600 nm^2^ toward the simulation end, consistent with receptor expansion and increased solvent accessibility during activation.

Moving to the GHSR complex, in RMSD analysis (Figure S5a), bromocriptine bound to GHSR stabilized around ~0.85 nm after ~15 ns, showing slightly lower fluctuation than the antagonist JMV-2959 (~1.0–1.2 nm) but higher stability than the agonist GHRP-6 (~0.9–1.0 nm). This suggests bromocriptine adopts a stable binding mode comparable to the antagonist but with marginally tighter RMSD convergence. RMSF patterns (Figure S5b) revealed that bromocriptine exhibited fluctuation peaks in three critical ligand-binding regions, including Asp99–Glu124, Leu181–Pro200, and Phe286–Ser308, that closely matched those of JMV-2959. These fluctuations indicate disruption of hydrogen-bonding and stabilizing contacts within the orthosteric site, a hallmark of antagonist-like interference with GHSR activation. In contrast, the agonist GHRP-6 showed dampened flexibility in these regions, supporting its role in stabilizing the receptor in an active conformation. RoG profiles (Figure S5c) showed bromocriptine maintained an average compactness of ~5.3 nm, higher than the agonist (~3.8 nm) but similar to the antagonist (~4.6–4.8 nm). This indicates that bromocriptine maintains a more open structural state relative to agonist binding, which may hinder conformational transitions needed for receptor activation. SASA values (Figure S5d) further supported this pattern, with bromocriptine displaying moderate solvent exposure (~150–250 nm^2^), lying between the antagonist (~200–300 nm^2^) and agonist (~100–200 nm^2^). The intermediate SASA profile suggests bromocriptine maintains partial receptor exposure to solvent, consistent with antagonist-like stabilization.

For OXTR (Figure S6a), RMSD analysis revealed that bromocriptine displayed higher structural fluctuations (~1.3–1.5 nm) compared to both the antagonist atosiban (~0.85–0.9 nm) and agonist oxytocin (~0.95–1.1 nm). This elevated RMSD suggests that bromocriptine binding may induce greater conformational rearrangements in OXTR, potentially destabilizing the active-state conformation. RMSF profiles (Figure S6b) showed that bromocriptine caused enhanced residue mobility in two key ligand-binding segments (Arg232–Leu265 and Ile280–Ala318) closely paralleling antagonist atosiban’s fluctuation patterns. This similarity points to disruption of critical hydrogen-bonding networks within the ligand-binding domain, limiting the structural rigidity required for agonist-mediated receptor activation. In contrast, oxytocin binding reduced flexibility in these residues, favoring activation. RoG analysis (Figure S6c) revealed that bromocriptine maintained a larger average radius (~5.8–6.2 nm) than both agonist (~5.2 nm) and antagonist (~4.5 nm), suggesting it induces a more expanded conformation. Such expansion is often linked with the destabilization of active-state packing. SASA measurements (Figure S6d) showed bromocriptine markedly increased solvent exposure (~300–600 nm^2^), particularly in the latter half of the simulation, while the antagonist atosiban remained consistently low (~150–200 nm^2^) and oxytocin showed a moderate increase (~200–350 nm^2^). The high SASA profile of bromocriptine suggests reduced burial within the OXTR binding site, which can hinder receptor activation efficiency. The complete MD simulation results can be seen in Supplementary Data S7.

**Figure 9:**
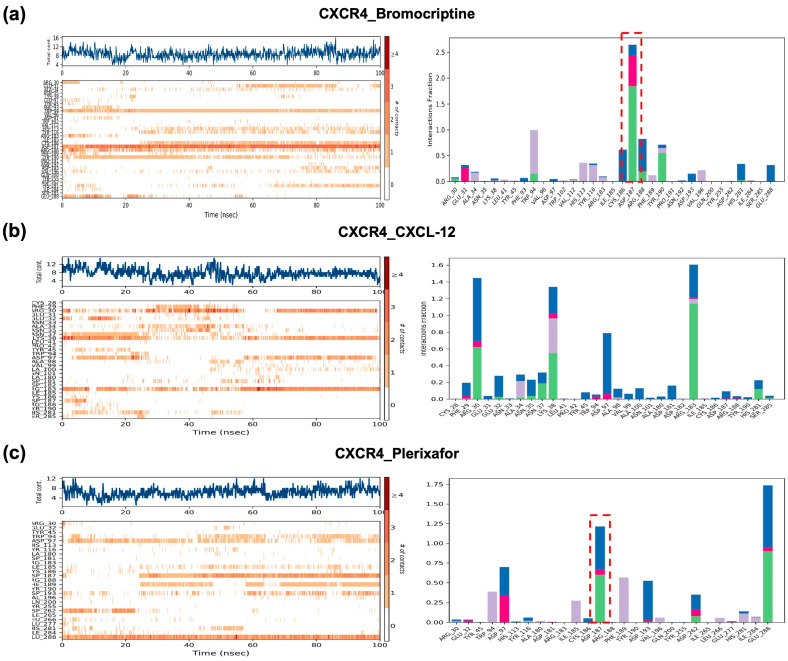
Histogram illustrating the number of contacts between CXCR4 and each studied ligand throughout the 100 ns MD simulation. (**a**) CXCR4_Bromocriptine complex. (**b**) CXCR4_CXCL-12 (agonist) complex. (**c**) CXCR4_Plerixafor (antagonist) complex. Bar colors represent different interaction types: blue = hydrogen bonds, green = hydrophobic contacts, pink = water bridges, purple = ionic interactions. The red dashed boxes highlight residues showing persistent and dominant interactions (e.g., Arg188 and Asp187) critical for ligand stabilization and help distinguish antagonist-like from agonist-like binding modes.

To characterize the molecular interactions between CXCR4 and its ligands, contact frequency histograms and interaction maps were generated throughout the 100 ns MD simulations for bromocriptine, the reference agonist CXCL-12, and the reference antagonist plerixafor (Figure 9). These analyses provide insights into which residues in the LBD contribute most consistently to ligand stabilization and whether bromocriptine preferentially mimics agonist- or antagonist-like contact patterns. For the CXCR4–bromocriptine complex (Figure 9a), the interaction heatmap and histogram reveal stable and recurrent contacts with several key residues in the LBD, most notably Asp187, Glu288, and His113. Asp187 stands out with an interaction fraction exceeding 2.0, indicating persistent and high-frequency contacts across the simulation trajectory. This is particularly noteworthy because Asp187 is a critical residue for antagonist binding in CXCR4, often involved in ionic or hydrogen-bond interactions that stabilize inactive conformations. The intense Asp187 contact in bromocriptine mirrors that observed for plerixafor, suggesting that bromocriptine engages the receptor in an antagonist-like manner at this site.

In contrast, the CXCR4–CXCL-12 agonist complex (Figure 9b) displayed a markedly different contact pattern. While Asp187 contacts were weaker and less frequent, strong interactions were concentrated in other extracellular loops and N-terminal residues, reflecting the typical engagement profile of peptide agonists. This shift in contact localization is consistent with agonist-induced receptor activation, which generally involves dynamic rearrangements in the LBD and transmembrane regions to facilitate signal transduction. For the CXCR4–plerixafor antagonist complex (Figure 9c), the contact profile exhibited pronounced and persistent interactions with Asp187, with an interaction fraction closely matching that seen in bromocriptine. Additional shared contacts included Glu288 and Tyr45, further supporting the similarity between bromocriptine and plerixafor in their binding orientation and stabilization strategy. The alignment of these interaction hotspots, particularly in Asp187, strongly indicates that bromocriptine preferentially stabilizes CXCR4 in an inactive conformation.

The MM/PBSA free binding energy calculations provide a quantitative measure of the thermodynamic stability of each ligand–receptor complex. Across all three receptors (CXCR4, GHSR, and OXTR), bromocriptine consistently demonstrated substantially favorable binding free energies, falling closer to those of the reference antagonists than to the agonists (Table 4). This trend reinforces the hypothesis that bromocriptine may preferentially adopt antagonist-like binding modes. For the CXCR4 complexes, bromocriptine achieved a binding free energy of −22.67 ± 3.70 kcal/mol, markedly stronger than the agonist CXCL-12 (−13.33 ± 4.62 kcal/mol) but somewhat weaker than the antagonist plerixafor (−28.43 ± 2.11 kcal/mol). This suggests that while bromocriptine does not reach the extreme binding affinity of plerixafor, it engages the receptor with energetics that are more characteristic of an antagonist.

In the GHSR complexes, bromocriptine bound with −22.11 ± 3.55 kcal/mol, again significantly stronger than the agonist GHRP-6 (−14.96 ± 4.78 kcal/mol) and only moderately weaker than the antagonist JMV-2959 (−24.98 ± 3.12 kcal/mol). This similarity to the antagonist profile supports the molecular dynamics contact analysis, which indicated overlap in binding site engagement patterns. For the OXTR complexes, bromocriptine displayed the least differential but still followed the same trend. Its binding free energy (−21.43 ± 2.41 kcal/mol) was stronger than that of the agonist oxytocin (−16.56 ± 4.72 kcal/mol) and close to that of the antagonist atosiban (−19.87 ± 3.21 kcal/mol). Interestingly, in OXTR, the bromocriptine antagonist energy difference was narrower, suggesting potentially more balanced binding characteristics between agonist and antagonist modes in this receptor context. Thus, MM/PBSA results strongly align with the molecular interaction profiles observed in the contact analysis. This indicates that bromocriptine generally exhibits antagonist-like binding energetics across all studied GPCR targets, with particularly strong alignment to known antagonists in CXCR4.

## 3. Discussion

This study presents a comprehensive computational exploration of bromocriptine, a clinically established dopamine D_2_ receptor agonist, focusing on its potential interactions with histamine-associated receptor targets. The work began by mapping bromocriptine-related proteins within histamine-enriched KEGG pathways, revealing a broad network of neuroactive ligand–receptor interactions. Among these, histamine receptor family members and several histamine-adjacent GPCRs emerged as network hubs. CXCR4, GHSR, and OXTR were prioritized for detailed evaluation from the enriched panel, molecular docking, and pharmacophore modeling. These receptors were subjected to an integrative molecular simulation pipeline combining MD simulations and MM/PBSA free energy calculations. Network pharmacology highlighted a substantial overlap between bromocriptine-associated proteins and histamine-related targets, with hubs enriched for GPCR signaling, PI3K–Akt, cAMP, and Ca^2+^ pathways and clustering in the “neuroactive ligand–receptor interaction” KEGG module. These same modules underlie transmitter and neuromodulator control in basal ganglia and limbic circuits, where histamine–dopamine interactions are well documented (e.g., H_3_R control of dopamine release and histaminergic contributions to neuroinflammation) [43,44]. Therefore, our pathway enrichments agree with prior mechanistic reviews that place histamine receptors at the neurotransmission and immune regulation interface.

Docking, pharmacophores, MD, and MM/PBSA consistently suggested that bromocriptine binds CXCR4, GHSR, and OXTR in more “antagonist-like” ways than “agonist-like.” For CXCR4, bromocriptine reproduced interaction hotspots around acidic residues that are known to anchor multiple antagonists. Crystal structures and mutational work have long implicated Asp97, Asp187, and Glu288 in small-molecule and peptide antagonist binding (e.g., IT1t and CVX15 in the seminal CXCR4 structures), and our models recapitulated this triad, with MD contact histograms emphasizing Asp187, mirroring the reference antagonist plerixafor [45,46]. For the GHSR, bromocriptine showed docking and MM/PBSA energies close to the known antagonist JMV-2959 and stronger than the peptide agonist GHRP-6, with pharmacophores capturing the same hydrophobic anchor regions and a polar feature near the Arg102/Gln120 pocket described in GHSR ligand studies. These patterns align with the established pharmacology of JMV-2959 as a competitive ghrelin receptor blocker and GHRP-6 as an agonist [47,48]. At OXTR, bromocriptine formed key contacts overlapping those used by small-molecule/peptidic antagonists (atosiban). It produced MD signatures (higher RMSF in binding loops, larger SASA and RoG) consistent with antagonist-like disruption rather than agonist stabilization by oxytocin. The structural literature on OXTR supports such interpretations: recent high-resolution complexes reveal how antagonists stabilize inactive conformations by engaging basic residues (e.g., Lys116) and hydrophobic subpockets that differ from oxytocin’s activation-biased pose [49].

Over 100 ns, bromocriptine–CXCR4 tracked the antagonist (plerixafor) rather than the agonist (CXCL-12) across four orthogonal MD readouts: (i) RMSD stabilization early and tightly; (ii) RMSF elevations at Trp94–Tyr116, Arg183–Tyr190, and Val280–Ile300 (consistent with partial H-bond network disruption required for activation); (iii) compact RoG; and (iv) lower SASA. Contact histograms localized persistent interactions to Asp187/Glu288, again echoing antagonist benchmarks. Similar, though receptor-specific, patterns were seen in GHSR (bromocriptine closer to JMV-2959 than to GHRP-6) and in OXTR (bromocriptine perturbing activation-linked regions more like atosiban). These dynamic behaviors match what is known from GPCR structural pharmacology: agonists typically reduce local flexibility in activation microswitches and increase global expansion during active-state formation, whereas antagonists preserve compactness and elevate local loop mobility that frustrates productive rearrangements [50,51].

An important limitation to acknowledge lies in the pharmacokinetic–pharmacodynamic (PK–PD) context. The actual in vivo plasma concentrations of bromocriptine may not reach the levels required to engage CXCR4, GHSR, or OXTR with the affinities predicted (ΔG_binding ~ −21 to −23 kcal/mol). Bromocriptine undergoes extensive first-pass metabolism and exhibits low systemic bioavailability, typically resulting in plasma concentrations in the low nanomolar range after therapeutic dosing [52,53]. Whether such exposure is sufficient to modulate these histamine-associated GPCRs remains an open question. Nevertheless, local tissue concentrations, particularly in the brain or pituitary, where bromocriptine is known to accumulate, may be higher than plasma levels and could allow engagement of secondary targets. These PK–PD considerations highlight the need for experimental studies that not only validate binding but also assess whether physiologically achievable drug concentrations translate into functional receptor modulation.

If borne out experimentally, antagonist-like modulation of CXCR4, GHSR, and OXTR by bromocriptine could intersect several histamine-relevant pathobiologies. CXCR4 is a central chemokine receptor in neuroinflammation and leukocyte trafficking; small-molecule antagonism (as with plerixafor) is clinically exploited for stem-cell mobilization and has been explored in neuroimmune contexts [54,55]. GHSR antagonism reduces ghrelin signaling, influencing dopaminergic tone, reward, and neuroinflammation; JMV-2959 has been widely used as a tool compound in those pathways [56,57]. OXTR antagonism modulates neuropeptide control of stress and social behavior and can influence glial and vascular responses [58]. Taken together, our computational findings, tempered by PK–PD considerations, suggest that bromocriptine’s polypharmacology could contribute to pleiotropic clinical effects, such as anti-inflammatory, metabolic, or stress-modulating outcomes, but careful experimental and pharmacological validation is essential before drawing translational conclusions. Given the literature linking histamine receptors to dopamine release and microglial activation [12,59], bromocriptine’s multi-receptor footprint might help explain reports of anti-inflammatory or disease-modifying signals beyond pure D_2_R agonism. These results generate specific, experimentally actionable hypotheses about bromocriptine’s polypharmacology at the interface of neurotransmission and inflammation, and they encourage prospective testing of this well-known drug in histamine- and chemokine-modulated indications.

## 4. Materials and Methods

### 4.1. Database Collection

Potential drug targets of bromocriptine were systematically retrieved from multiple sources, including the Similarity Ensemble Approach (SEA) [52] (https://sea.bkslab.org/ (accessed on 14 April 2025), DrugBank [53] (https://go.drugbank.com/ (accessed on 14 April 2025)), Search Tool for Interacting Chemicals (STITCH) [60] (http://stitch.embl.de/ (accessed on 14 April 2025)), PharmMapper [61] (https://www.lilab-ecust.cn/pharmmapper/ (accessed on 14 April 2025)), and TargetNet [62] (http://targetnet.scbdd.com/(accessed on 14 April 2025)). The PubChem database [63] (https://pubchem.ncbi.nlm.nih.gov/ (accessed on 14 April 2025)) was used to obtain the Simplified Molecular Input Line Entry System (SMILES) representation of bromocriptine, which was subsequently queried in SwissTargetPrediction (STP) [64] (http://www.swisstargetprediction.ch/ (accessed on 14 April 2025)) to predict additional putative targets. The prediction was restricted to Homo sapiens proteins, applying a Tanimoto Coefficient (TC) cutoff of ≥0.50. This value was chosen to balance sensitivity and specificity, since higher cutoffs yield fewer predicted targets but enhance the reliability of the predicted associations [65]. All retrieved bromocriptine-associated targets were standardized to official gene symbols using the UniProt database [66] (https://www.uniprot.org/ (accessed on 14 April 2025)), and duplicate entries were removed to yield a non-redundant target list. Histamine-associated targets were collected from GeneCards [67] (https://www.genecards.org/ (accessed on 14 April 2025)), Online Mendelian Inheritance in Man (OMIM) [68] https://omim.org/ (accessed on 14 April 2025)), and NCBI Gene (https://www.ncbi.nlm.nih.gov/gene/ (accessed on 14 April 2025)), with the search restricted to Homo sapiens. Redundant entries were eliminated to establish a comprehensive histamine target library. The intersection between bromocriptine and histamine-associated targets was determined and visualized using a Venn diagram, representing the overlap as potential histamine-related targets of bromocriptine. This intersection set was the foundation for subsequent network pharmacology, pathway enrichment, and structural analysis.

### 4.2. Protein–Protein Interaction (PPI) Network Construction

The intersecting gene set between bromocriptine and histamine-associated targets was analyzed to explore potential PPIs using the STRING database [69] (https://string-db.org/ (accessed on 18 April 2025)). The query was restricted to Homo sapiens and a minimum required interaction score of 0.40 (medium confidence) [21]. The resulting interaction network was exported for further analysis. Topological analysis of the PPI network was performed using Cytoscape version 3.10.3 [70] (https://www.cytoscape.org/ (accessed on 18 April 2025)) with the CytoNCA plugin [71]. Four key topological parameters were calculated to assess the centrality and importance of each node: DC, EC, BC, and CC. Nodes with values meeting or exceeding the respective median thresholds across all four parameters were designated as hub nodes, representing potential core targets. In the visualization, node color intensity was scaled to reflect the relative topological importance, with darker hues indicating higher centrality.

### 4.3. Gene Ontology (GO) and Kyoto Encyclopedia of Genes and Genomes (KEGG) Enrichment Analyses

To elucidate the potential biological functions and signaling pathways associated with bromocriptine’s interaction with histamine-related targets, a network enrichment analysis was performed. In this context, “network enrichment analysis” refers to a statistical approach used to determine whether specific biological processes, molecular functions, cellular components, or signaling pathways are represented more frequently than expected by chance within the set of overlapping bromocriptine–histamine-associated targets. By mapping these targets onto curated ontologies and pathway databases, the method highlights biological modules or signaling cascades that are disproportionately represented, thereby identifying functional themes that may underlie bromocriptine’s pharmacological effects. For this purpose, GO annotations were retrieved from the Gene Ontology database [72] (https://geneontology.org/ (accessed on 22 April 2025)), while KEGG pathway data were obtained from the KEGG resource [73] (https://www.genome.jp/ (accessed on 22 April 2025)). Enrichment analyses were conducted using the ShinyGO 0.82 [74] web-based platform (https://bioinformatics.sdstate.edu/go/ (accessed on 22 April 2025)), covering the three GO categories: BP, MF, and CC, as well as KEGG signaling pathways. Statistical significance was determined using a false discovery rate (FDR) < 0.05 and *p* < 0.05. The top 10 enriched GO terms in each category and the top 10 KEGG pathways were selected for visualization. These results were illustrated through bar plots, network diagrams, and hierarchical clustering to highlight functional associations, pathway interconnectivity, and fold enrichment patterns.

### 4.4. Molecular Docking Simulations and Binding Affinity Analysis

Molecular docking simulations were performed to elucidate the molecular basis of bromocriptine interaction with histamine-associated targets. The primary objectives were to identify key binding residues involved in ligand–receptor interactions, characterize the intermolecular forces contributing to binding affinity, and explore the binding modes and orientations of the compound in comparison with standard ligands. This approach aimed to provide structural insights into bromocriptine’s potential modulation of histamine-related signaling pathways. Three-dimensional (3D) structures of selected receptors identified through PPI network analysis and KEGG pathway enrichment were retrieved from the RCSB Protein Data Bank (PDB) (https://www.rcsb.org/ (accessed on 25 April 2025)). Prior to docking, receptor structures were refined using Swiss-PdbViewer version 4.1 [75] (Swiss Institute of Bioinformatics, Lausanne, Switzerland) to ensure optimal geometry. Refinement steps included energy minimization, removal of crystallographic water molecules, and reconstruction of missing side chains. Active site identification was conducted using PDBSum [76] (European Bioinformatics Institute, Cambridge, UK) (https://www.ebi.ac.uk/thornton-srv/databases/pdbsum/ (accessed on 25 April 2025)), which provides detailed structural summaries including ligand-binding pockets and secondary structure features. This ensured docking simulations targeted biologically relevant binding regions. Bromocriptine molecular structure was generated and energy-minimized using the molecular mechanics 2 (MM2) force field [77] in Chem3D Ultra version 22 (PerkinElmer, Waltham, MA, USA). This step optimized the molecular geometry to the lowest energy conformation before docking. Reference ligands known to interact with the respective receptors were included as controls. These standard ligands underwent identical MM2 energy minimization procedures to maintain methodological consistency across docking experiments.

Molecular docking was performed using the High Ambiguity Driven Protein–Protein Docking (HADDOCK) version 2.4 (University of Utrecht, Utrecht, The Netherlands) (https://wenmr.science.uu.nl/haddock2.4/ (accessed on 25 April 2025)). HADDOCK employs ambiguous interaction restraints (AIRs) derived from experimental or predicted binding site information, improving docking accuracy [78,79]. The standalone HADDOCK interface enabled advanced customization of docking parameters, integrating both geometric and energetic constraints to enhance the reliability of the predicted complexes. Docking results were evaluated using two primary criteria. The first was cluster population, in which docking models were grouped into clusters, with the most populated cluster considered the most reproducible and stable binding mode. The second was the HADDOCK score, a composite metric incorporating van der Waals energy, electrostatic interactions, desolvation energy, and buried surface area. The docking model with the most favorable HADDOCK score was selected for subsequent analysis, representing the highest predicted binding affinity. PRODIGY (PROtein binDIng enerGY prediction) (https://wenmr.science.uu.nl/prodigy/ (accessed on 25 April 2025)) [80] was employed to refine free energy estimates. PRODIGY calculates binding free energy (ΔG, kcal/mol) based on the structural and energetic features of the ligand–receptor complex, providing an additional layer of validation to the docking results.

### 4.5. Pharmacophore Modeling

To identify the essential molecular features mediating the interaction between bromocriptine and histamine-associated target receptors, pharmacophore modeling was conducted using LigandScout 4.5 (Inte:Ligand, Vienna, Austria) [81]. LigandScout enables the generation of three-dimensional (3D) pharmacophore models by detecting and mapping critical interaction features, including hydrogen bond donors (HBD), hydrogen bond acceptors (HBA), hydrophobic regions, aromatic rings, and electrostatic interaction sites. These features are fundamental for ligand recognition and play a central role in determining binding specificity and affinity toward target proteins. The receptor–ligand complexes selected from the molecular docking stage were the basis for pharmacophore generation. Bromocriptine-bound receptor structures were imported into LigandScout version 4.4, and the software automatically extracted pharmacophoric features by analyzing spatial arrangements and interaction patterns within the binding pocket. This process involved identifying conserved hydrogen bonds, hydrophobic contacts, and π–π stacking interactions and mapping regions critical for electrostatic complementarity [82,83]. To improve model robustness and predictive accuracy, the pharmacophore features derived from bromocriptine were compared with those generated from reference ligands that bind the same receptors. This comparative analysis facilitated the selection of key chemical moieties likely responsible for the compound’s biological activity.

### 4.6. Molecular Dynamics (MD) Simulation for Structural Stability and Interaction Analysis

To evaluate the structural stability and dynamic behavior of the protein–ligand complexes, MD simulations were conducted using the Desmond MD simulation package integrated within the Schrödinger Suite (v12.5.139). This approach allowed for in-depth exploration of conformational flexibility, stability of molecular interactions, and time-dependent structural deviations of the complexes under physiological conditions. The OPLS3e force field was employed to ensure accurate representation of atomic interactions throughout the simulation process [84]. Each complex was initially prepared using a standardized protocol involving solvation, charge neutralization, energy minimization, equilibration, and production simulation. The protein–ligand systems were solvated using the TIP3P water model within an orthorhombic periodic boundary box, maintaining a 10 Å buffer distance between the complex and the box edges to prevent boundary artifacts. Sodium (Na^+^) and chloride (Cl^−^) counterions were added to neutralize the total system charge [85].

Following solvation and neutralization, energy minimization was performed to eliminate unfavorable steric clashes. This step was followed by a stepwise equilibration phase, consisting of 1 ns under the NVT ensemble (constant number of particles, volume, and temperature) and 1 ns under the NPT ensemble (constant number of particles, pressure, and temperature). The Nose–Hoover thermostat was applied to regulate temperature at 300 K, while the Martyna–Tobias–Klein barostat was used to maintain pressure at 1.01 bar, simulating realistic biological conditions [86,87]. The production MD simulations were carried out for 100 ns under NPT conditions. Throughout the simulation, trajectory data were collected at regular intervals and analyzed using Desmond’s built-in tools. Key evaluation metrics included RMSD to monitor global structural stability, RMSF to assess residue-level flexibility, and detailed protein–ligand interaction profiles over time. These interaction analyses focused on hydrogen bonds, hydrophobic interactions, salt bridges, and π-π stacking, providing insight into the persistence and stability of binding modes throughout the simulation period.

### 4.7. MM/PBSA Binding Free Energy Calculation Using gmx_MMPBSA

The Molecular Mechanics/Poisson–Boltzmann Surface Area (MM/PBSA) method was applied using gmx_MMPBSA v1.6.4 [88] to estimate the binding free energy of protein-ligand complexes. Since the original MD simulations were performed using Desmond (Schrödinger Suite v12.5.139), the resulting trajectories (.dtr) and structural files (.cms) were first exported to PDB format via Maestro. These were converted into GROMACS-compatible formats (.xtc, .tpr, .top) using VMD, ACPYPE, and GROMACS v2025.1 utilities [89,90,91]. Ligand topology files were generated using Antechamber with the GAFF force field, while the full system topology was assembled by merging protein and ligand components. An index file was created using gmx make_ndx to define the complex, protein, and ligand groups for downstream analysis. The MM/PBSA analysis was performed using 100 snapshots evenly extracted from the 100 ns MD trajectory, excluding water and ions. The input file (mmpbsa.in) was configured to apply the Poisson–Boltzmann solvation model, and the calculation was executed with the standard gmx_MMPBSA command. The final binding free energy (ΔG_bind) was computed as the sum of molecular mechanics energy (van der Waals and electrostatics), polar solvation energy, and nonpolar solvation energy based on solvent-accessible surface area (SASA). Entropic contributions (TΔS) were omitted due to computational limitations. Output files included the total ΔG_bind, energy decomposition data, and per-residue free energy contributions, which were used to identify key interacting residues and evaluate the binding strength of each complex.

## 5. Limitations, Clinical Implications, and Future Works

Several limitations should be acknowledged when interpreting these findings. First, the study is entirely computational, relying on network pharmacology, molecular docking, pharmacophore modeling, MD simulations, and MM/PBSA binding energy calculations. While these approaches provide valuable mechanistic insights, they cannot fully replicate the complexity of in vivo biological systems. Receptor conformational flexibility, membrane microenvironment effects, and the influence of co-factors or interacting proteins may alter bromocriptine’s binding modes in physiological conditions. Furthermore, our analysis focused on static receptor structures or MD-simulated conformations of selected GPCRs, potentially overlooking dynamic allosteric sites or ligand-induced conformational states that could influence functional outcomes. Finally, the network pharmacology component was constrained by available databases, which may contain incomplete or biased interaction data. In particular, the absence of experimental validation (e.g., receptor binding or competition assays) limits the ability to directly confirm the predicted interactions, and this is acknowledged as an essential next step.

We acknowledge that the ~100 ns MD trajectories employed here represent a relatively short timescale compared with the microsecond-scale simulations commonly used to capture large-scale conformational rearrangements in GPCRs. Our primary objective, however, was not to resolve slow allosteric transitions but to validate docking poses and assess local binding stability. Within 100 ns, the RMSD, RMSF, RoG, and hydrogen-bonding analyses reached stable plateaus, supporting equilibration of the complexes and robustness of the proposed binding modes. Importantly, subsequent MM/PBSA free energy calculations converged consistently across replicates, further reinforcing stability on this timescale. In addition, docking itself was performed using a semi-flexible HADDOCK protocol, which allowed limited side-chain adjustments during complex formation and reduced dependence on a purely rigid force-field representation. We fully acknowledge that the absence of steered molecular dynamics (SMD) force-pulling simulations limits the direct mechanical validation of the proposed binding poses, and that extending MD trajectories to the microsecond regime would provide deeper insight into slower conformational events. These remain valuable directions for future work. Nevertheless, the combined use of semi-flexible docking, explicit-solvent MD equilibration, and MM/PBSA free energy calculations provides a widely accepted framework for pose validation, mitigating force-field dependence and supporting the reliability of our conclusions.

Despite these constraints, the findings carry essential potential clinical implications. Bromocriptine is primarily prescribed as a dopamine D_2_ receptor agonist, yet our data suggest it may also engage in both antagonist-like and mixed interaction modes with histamine-associated GPCRs such as CXCR4, GHSR, and OXTR. This polypharmacology could explain specific pleiotropic clinical effects of bromocriptine, including modulation of neuroinflammation, appetite regulation, stress responses, and possibly vascular tone. CXCR4 antagonism, for example, has been implicated in reducing neuroinflammatory cascades and influencing immune cell trafficking, which could extend bromocriptine’s therapeutic utility beyond its dopaminergic profile. Similarly, modulation of GHSR may contribute to metabolic regulation, while OXTR interaction may influence psychosocial and stress-related behaviors. Understanding these off-target activities may inform safer dosing strategies, drug repurposing efforts, and personalized medicine approaches, particularly in patients with comorbid neurological and inflammatory conditions.

Future studies should validate these computational predictions through experimental approaches, including competition binding assays in living cells, in vitro receptor binding assays, cell-based functional assays, and ultimately in vivo pharmacological evaluations. High-resolution cryo-EM or X-ray crystallography of bromocriptine-bound CXCR4, GHSR, and OXTR could clarify the structural basis of its observed antagonist-like and modulatory binding modes. Additionally, given the role of histamine-related pathways in diverse physiological and pathological processes, transcriptomic or proteomic studies in relevant disease models could help elucidate the downstream effects of bromocriptine’s multi-receptor engagement. From a translational perspective, these insights may guide the development of novel bromocriptine derivatives or analogs with optimized selectivity profiles, allowing therapeutic targeting of specific GPCR subsets while minimizing unwanted effects. Expanding the scope to include other histamine-linked GPCRs and integrating patient-derived organoid models or induced pluripotent stem cell (iPSC) systems could further bridge the gap between computational predictions and clinical application.

## 6. Conclusions

In conclusion, this study provides molecular-level insights into bromocriptine’s interactions with histamine-associated GPCR targets, identified through network pharmacology as CXCR4, GHSR, and OXTR, and validated by docking, pharmacophore modeling, and molecular dynamics simulations. Bromocriptine consistently demonstrated stable binding orientations, persistent residue contacts, and favorable binding free energies, aligning more closely with antagonist reference ligands yet also exhibiting unique interaction features that suggest broader modulatory potential. The convergence of structural stability, dynamic behavior, and energetics underscores bromocriptine’s capacity to engage histamine-related receptors in a manner that could influence both dopaminergic and histaminergic signaling. Collectively, these findings highlight bromocriptine’s potential as a multi-target therapeutic agent and provide a computational framework for its repurposing in histamine-associated disorders, while warranting further experimental validation to confirm its functional pharmacology.

## Figures and Tables

**Table 1:** Top 10 bromocriptine–receptor complexes ranked by HADDOCK score, with associated binding free energies and interaction energy components.

Complex	HADDOCK Score (a.u.)	Binding Energy (kcal/mol)	Van der Waals Energy	Electrostatic Energy	Desolvation Energy	RMSD
CXCR4_Bromocriptine	−56.1 ± 0.7	−10.67	−36.9 ± 0.6	−124.0 ± 9.1	−7.2 ± 0.4	0.2 ± 0.1
GHSR_Bromocriptine	−62.9 ± 1.6	−10.36	−39.4 ± 1.5	−54.3 ± 7.5	−18.4 ± 0.7	0.3 ± 0.2
DRD2_Bromocriptine	−57.4 ± 0.5	−10.29	−35.5 ± 1.4	−71.5 ± 7.3	−14.9 ± 0.6	0.2 ± 0.2
OXTR_Bromocriptine	−56.5 ± 0.9	−9.93	−32.0 ± 0.4	−21.3 ± 7.3	−22.8 ± 1.3	0.4 ± 0.2
DRD3_Bromocriptine	−52.8 ± 0.3	−9.77	−30.3 ± 0.3	−71.8 ± 5.0	−15.4 ± 0.5	0.3 ± 0.2
HTR1A_Bromocriptine	−52.6 ± 0.1	−9.50	−33.0 ± 0.6	−53.3 ± 1.9	−14.4 ± 0.4	0.3 ± 0.2
ERBB2_Bromocriptine	−42.5 ± 1.7	−9.37	−32.5 ± 0.8	−79.5 ± 8.4	−3.3 ± 0.4	0.6 ± 0.0
HRH3_Bromocriptine	−56.7 ± 0.2	−9.15	−37.3 ± 0.4	−84.0 ± 3.6	−11.1 ± 0.4	0.3 ± 0.2
TSHR_Bromocriptine	−40.9 ± 2.9	−9.08	−31.0 ± 1.1	−76.0 ± 6.6	−6.7 ± 0.2	0.3 ± 0.0
EGFR_Bromocriptine	−43.8 ± 1.0	−9.02	−35.7 ± 1.6	−36.7 ± 5.8	−5.1 ± 1.2	0.5 ± 0.0

**Table 2:** Molecular interaction profiles between bromocriptine and target proteins based on atom–atom contact types.

Complex	CC	CO	CN	CX	OO	OX	NO	NN	NX	XX
CXCR4_Bromocriptine	3179	1357	1366	35	133	2	267	132	4	0
GHSR_Bromocriptine	3658	1545	1414	47	162	9	283	126	8	0
DRD2_Bromocriptine	3298	1504	1212	58	149	10	270	100	9	0
OXTR_Bromocriptine	3376	1234	1164	43	111	12	193	90	7	0
DRD3_Bromocriptine	2949	1302	1150	32	131	1	239	112	5	0
HTR1A_Bromocriptine	3015	1216	1189	62	115	10	242	120	14	0
ERBB2_Bromocriptine	2719	1172	1183	44	101	5	218	121	6	0
HRH3_Bromocriptine	2468	936	1091	27	83	0	196	108	4	0
TSHR_Bromocriptine	2371	1252	892	14	145	5	233	84	3	0
EGFR_Bromocriptine	2919	1260	1321	55	113	4	263	142	9	0

Note: CC: Carbon–carbon interaction; CO: Carbon–oxygen interaction; CN: Carbon–nitrogen interaction; CX: Carbon–any heavy atom interaction; OO: Oxygen–oxygen interaction; OX: Oxygen–any heavy atom interaction; NO: Nitrogen–oxygen interaction; NN: Nitrogen–nitrogen interaction; NX: Nitrogen–any heavy atom interaction; XX: Any heavy–heavy atom interaction.

**Table 3:** Comparative molecular docking results of bromocriptine compared to standard agonists and antagonists in CXCR4, GHSR, and OXTR.

Complex	HADDOCK Score (a.u.)	Binding Energy (kcal/mol)	Van der Waals Energy	Electrostatic Energy	Desolvation Energy	RMSD
CXCR4_Bromocriptine	−56.1 ± 0.7	−10.67	−36.9 ± 0.6	−124.0 ± 9.1	−7.2 ± 0.4	0.2 ± 0.1
CXCR4_CXCL-12 (agonist)	−21.0 ± 2.6	−7.06	−17.3 ± 2.2	−62.2 ± 21.1	1.2 ± 0.5	0.5 ± 0.0
CXCR4_Plerixafor (antagonist)	−79.7 ± 4.2	−14.21	−26.1 ± 1.8	−530.0 ± 19.2	−3.3 ± 0.4	0.5 ± 0.1
GHSR_Bromocriptine	−62.9 ± 1.6	−10.36	−39.4 ± 1.5	−54.3 ± 7.5	−18.4 ± 0.7	0.3 ± 0.2
GHSR_GHRP-6 (agonist)	−48.6 ± 0.4	−8.58	−37.9 ± 1.2	−19.9 ± 20.4	−12.2 ± 1.5	0.7 ± 0.1
GHSR_JMV-2959 (antagonist)	−62.5 ± 1.3	−10.49	−36.3 ± 0.8	−89.8 ± 9.2	−17.5 ± 1.3	0.3 ± 0.2
OXTR_Bromocriptine	−56.5 ± 0.9	−9.93	−32.0 ± 0.4	−21.3 ± 7.3	−22.8 ± 1.3	0.4 ± 0.2
OXTR_Oxytocin (agonist)	−58.6 ± 1.3	−8.93	−35.9 ± 2.4	−29.7 ± 4.4	−21.6 ± 1.5	0.2 ± 0.1
OXTR_Atosiban (antagonist)	−56.5 ± 1.1	−8.99	−33.9 ± 0.7	−36.5 ± 9.8	−19.4 ± 0.7	0.6 ± 0.1

**Table 4:** Comparative MM/PBSA free binding energies of bromocriptine and reference agonists and antagonists with CXCR4, GHSR, and OXTR.

Complex	MM/PBSA Free Binding Energy ΔG_Binding (kcal/mol)
CXCR4_Bromocriptine	−22.67 ± 3.70
CXCR4_CXCL-12 (agonist)	−13.33 ± 4.62
CXCR4_Plerixafor (antagonist)	−28.43 ± 2.11
GHSR_Bromocriptine	−22.11 ± 3.55
GHSR_GHRP-6 (agonist)	−14.96 ± 4.78
GHSR_JMV-2959 (antagonist)	−24.98 ± 3.12
OXTR_Bromocriptine	−21.43 ± 2.41
OXTR_Oxytocin (agonist)	−16.56 ± 4.72
OXTR_Atosiban (antagonist)	−19.87 ± 3.21

## Data Availability

The original contributions presented in this study are included in the article/Supplementary Material. Further inquiries can be directed to the corresponding author(s).

## Supplementary Materials

The following supporting information can be downloaded at https://www.mdpi.com/article/10.3390/ijms26178717/s1.

## Abbreviations

The following abbreviations are used in this manuscript:

AIRs	Ambiguous interaction restraints
AKT1	AKT serine/threonine kinase 1
APP	Amyloid precursor protein
BC	Betweenness centrality
BP	Biological process
CASP3	Caspase-3
CC	Closeness centrality
CDS	Coding sequence
CXCR4	C-X-C chemokine receptor type 4
DC	Degree centrality
DRD2	D2 dopamine receptor
EC	Eigenvector centrality
EGFR	Epidermal growth factor receptor
FDA	Food and Drug Administration
FDR	False discovery rate
GABA	γ-aminobutyric acid
H_1_R	Histamine receptor H1
HADDOCK	High ambiguity driven protein-protein docking
HBA	Hydrogen bond acceptor
HBD	Hydrogen bond donor
ICAM1	Intercellular adhesion molecule 1
IGF1	Insulin-like growth factor 1
IL2	Interleukin-2
iPSC	Induced pluripotent stem cell
LBD	Ligand-binding domain
MD	Molecular dynamics
MF	Molecular function
MM/PBSA	Molecular mechanics/Poisson-Boltzmann surface area
MMP9	Matrix metalloproteinase-9
NPT	Number of particles, pressure, and temperature
NVT	Number of particles, volume, and temperature
OMIM	Online Mendelian inheritance in man
PDB	Protein data bank
PI3K	Phosphatidylinositol 3-kinase
PK–PD	Pharmacokinetic–pharmacodynamic
PPI	Protein–protein interaction
PRODIGY	Protein binding energy prediction
RMSD	Root mean square deviation
RMSF	Root mean square fluctuation
RoG	Radius of gyration
SASA	Solvent-accessible surface area
SEA	Similarity ensemble approach
SMD	Steered molecular dynamics
SMILES	Simplified molecular input line entry system
SRC	Proto-oncogene tyrosine-protein kinase Src
STITCH	Search tool for interacting chemicals
STP	SwissTargetPrediction
TC	Tanimoto coefficient
TNF	Tumor necrosis factor
