# Supplementary material for: Molecular Insights into Bromocriptine Binding to GPCRs Within Histamine-Linked Signaling Networks: Network Pharmacology, Pharmacophore Modeling, and Molecular Dynamics Simulation
Source: Int J Mol Sci. 2025 Sep 7;26(17):8717. doi: 10.3390/ijms26178717 (PMC12428908; doi:10.3390/ijms26178717)
Supplement: Supplementary file 1 [file ijms-26-08717-s001.zip › Supplementary Data S5 - 2D and 3D Interactions.pdf]

Supplementary File S5

2D and 3D Interactions of Top Ligand-Receptor Complexes

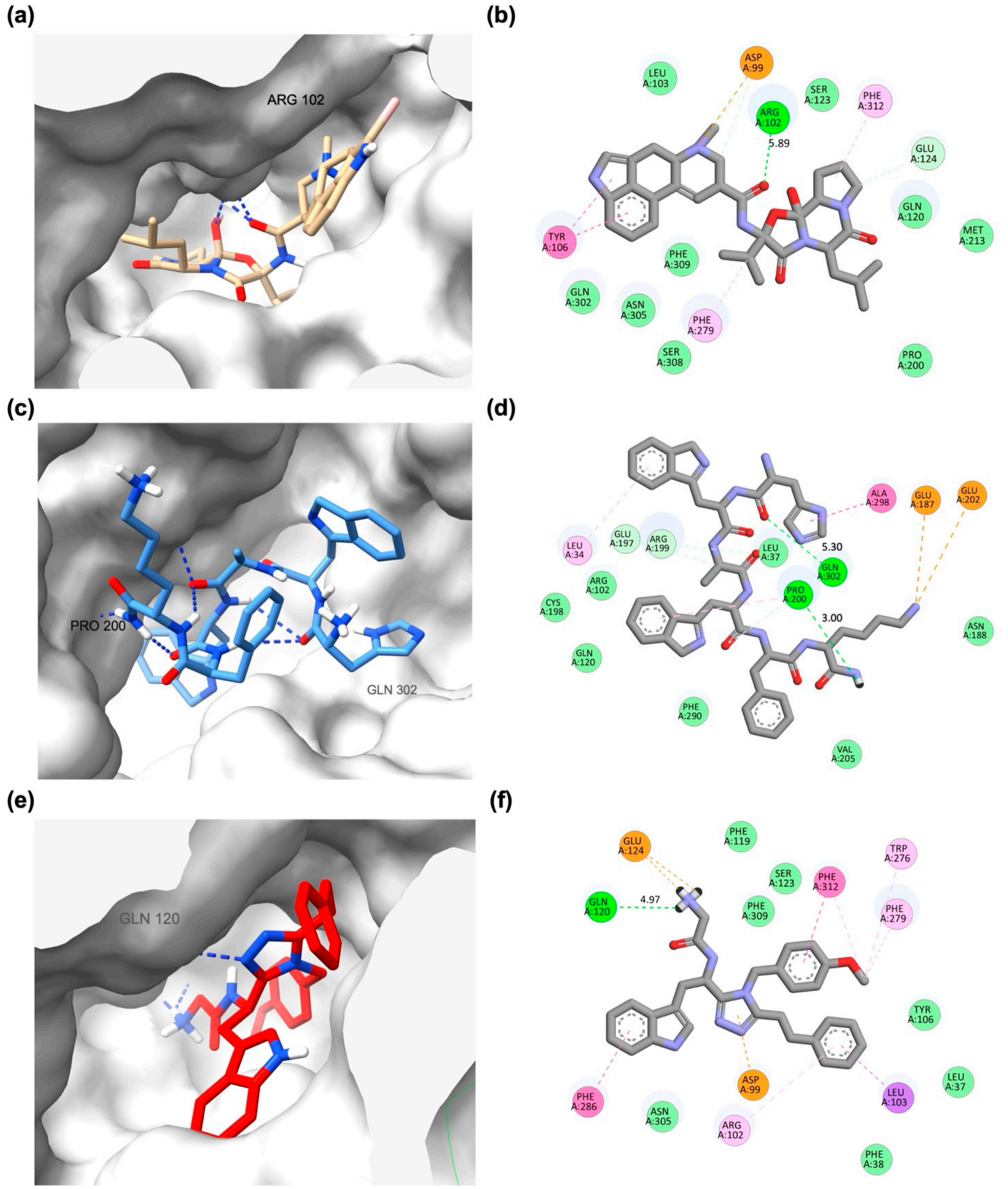

Figure S1. Comparative 3D and 2D binding pose of bromocriptine, standard agonists, and antagonists in the GHSR ligand-binding domain (LBD). (**a**) 3D binding pose of bromocriptine in GHSR. (**b**) 2D interaction map of bromocriptine–GHSR complex showing hydrogen bonds with Arg102. (**c**) 3D binding pose of GHRP-6 (agonist) in GHSR. (**d**) 2D interaction map of GHSR_GHRP-6 complex highlighting hydrogen bonds with Pro200 and Gln302. (**e**) 3D binding pose of JMV-2959 (antagonist) in GHSR. (**f**) 2D interaction map of JMV-2959–GHSR complex showing hydrogen bonding with Gln120. The

interaction types are color-coded as follows: hydrogen bonds (bright green), van der Waals interactions (pale green), Pi-Alkyl (pink), Pi-Sigma (purple), Pi-Sulfur (orange), and Halogen interactions (bright blue).

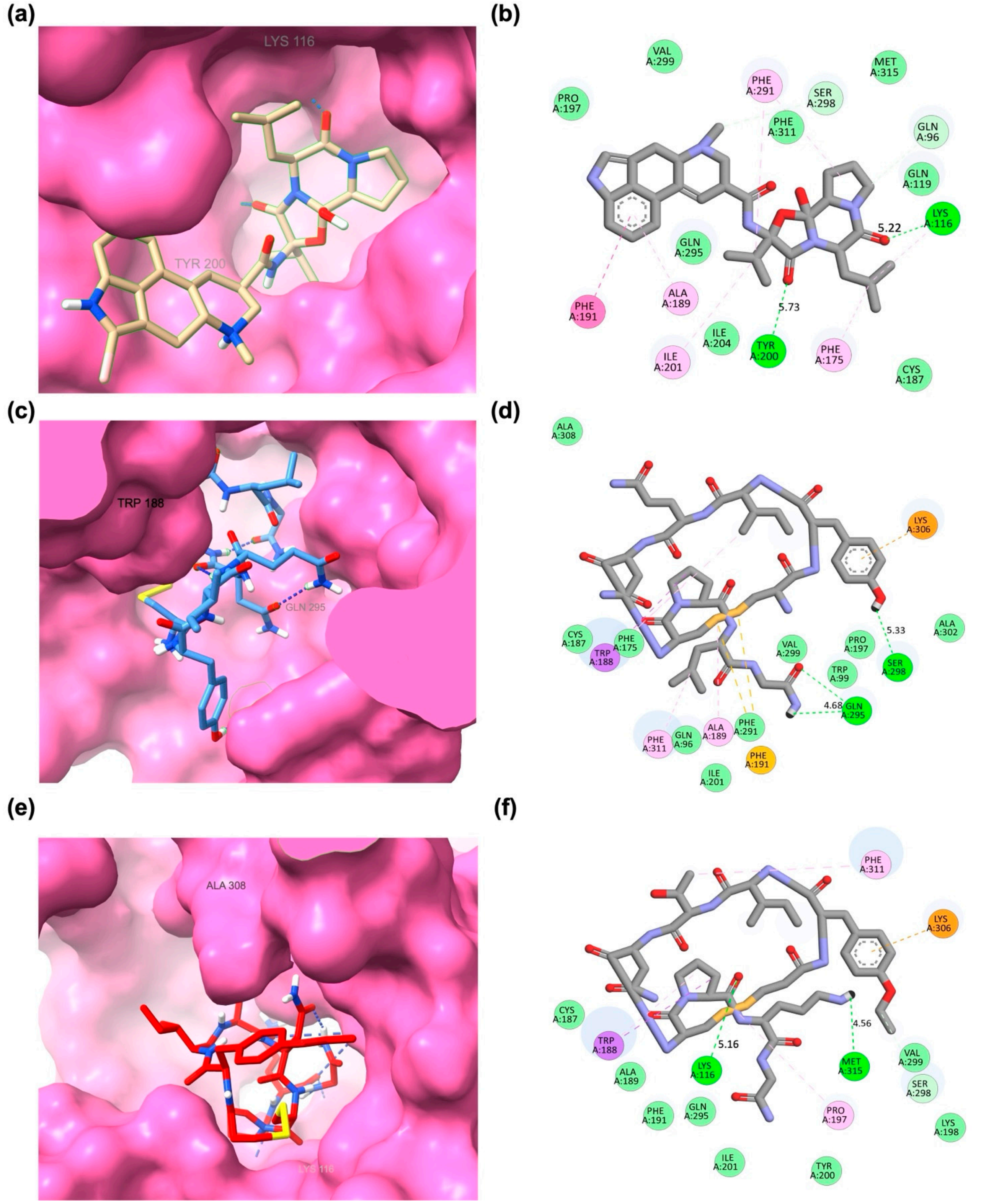

Figure S2. Comparative 3D and 2D binding pose of bromocriptine, standard agonists, and antagonists in the OXTR ligand-binding domain (LBD). (**a**) 3D binding pose of bromocriptine in OXTR. (**b**) 2D interaction map of bromocriptine–OXTR complex showing hydrogen bonds with Lys116 and Tyr200. (**c**) 3D binding pose of Oxytocin (agonist) in OXTR. (**d**) 2D interaction map of Oxytocin–OXTR complex highlighting hydrogen bonds and hydrophobic contacts. (**e**) 3D binding pose of Atosiban (antagonist) in OXTR. (**f**) 2D interaction map of Atosiban–OXTR complex showing hydrogen bonding with

Lys116 and Met315. The interaction types are color-coded as follows: hydrogen bonds (bright green), van der Waals interactions (pale green), Pi-Alkyl (pink), Pi-Sigma (purple), Pi-Sulfur (orange), and Halogen interactions (bright blue).
