# Supplementary material for: Molecular Insights into Bromocriptine Binding to GPCRs Within Histamine-Linked Signaling Networks: Network Pharmacology, Pharmacophore Modeling, and Molecular Dynamics Simulation
Source: Int J Mol Sci. 2025 Sep 7;26(17):8717. doi: 10.3390/ijms26178717 (PMC12428908; doi:10.3390/ijms26178717)
Supplement: Supplementary file 1 [file ijms-26-08717-s001.zip › Supplementary Data S6 - Pharmacophore Modeling.pdf]

Supplementary File S6

Pharmacophore Modeling of Top Ligand-Receptor Complexes

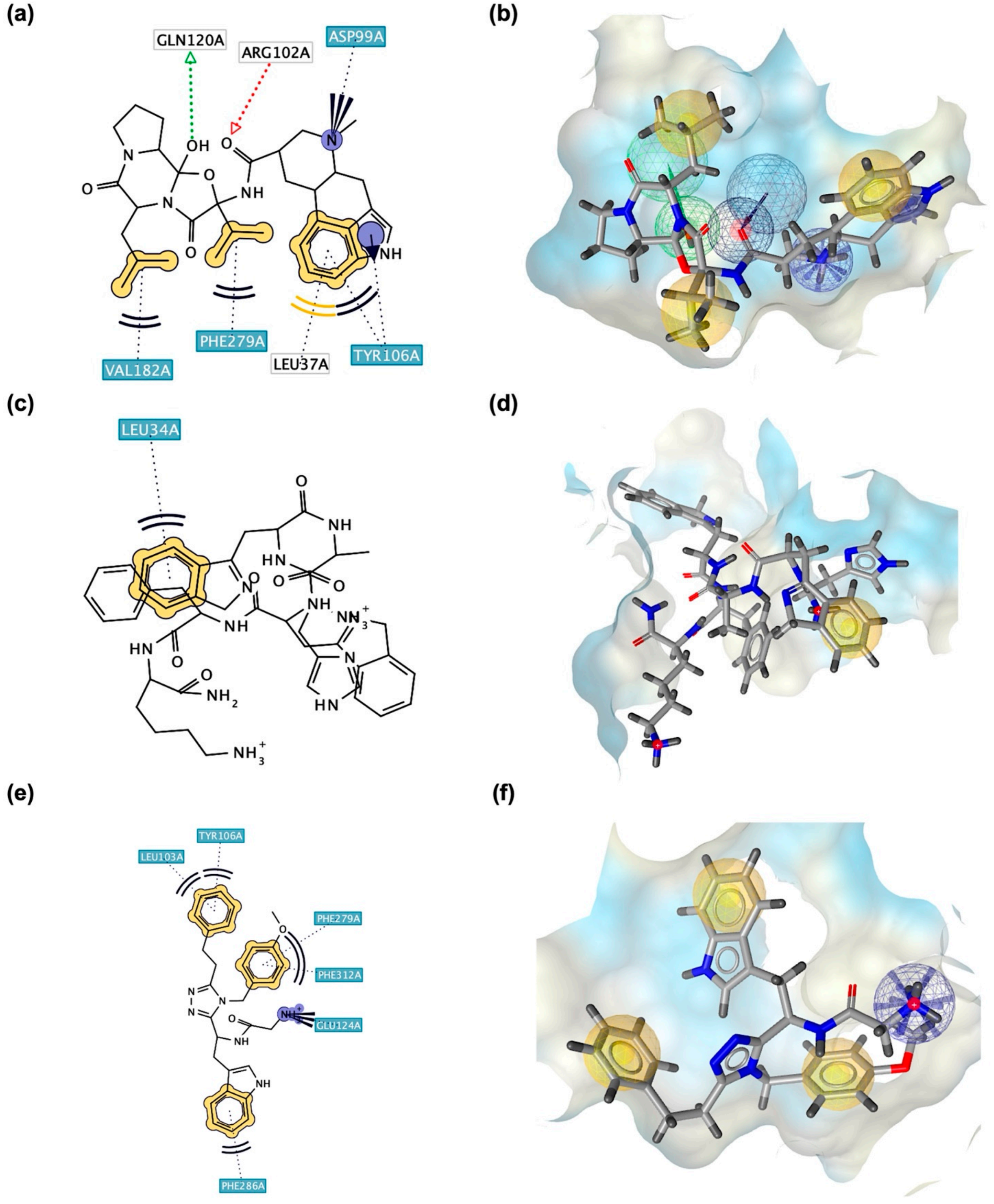

Figure S3. Pharmacophore modeling results of bromocriptine, standard agonists, and antagonists in the GHSR LBD. (**a**) 2D pharmacophore model of GHSR_Bromocriptine. (**b**) 3D pharmacophore model of GHSR_Bromocriptine. (**c**) 2D pharmacophore model of GHSR_GHRP-6 (agonist). (**d**) 3D pharmacophore model of GHSR_GHRP-6 (agonist). (**e**) 2D pharmacophore model of GHSR_JMV-2959 (antagonist). (**f**) 3D pharmacophore model of GHSR_JMV-2959 (antagonist). Yellow spheres indicate hydrophobic interactions, green arrows represent hydrogen bond donors, red arrows signify hydrogen bond acceptors, and blue stars represent a positive ionizable group.

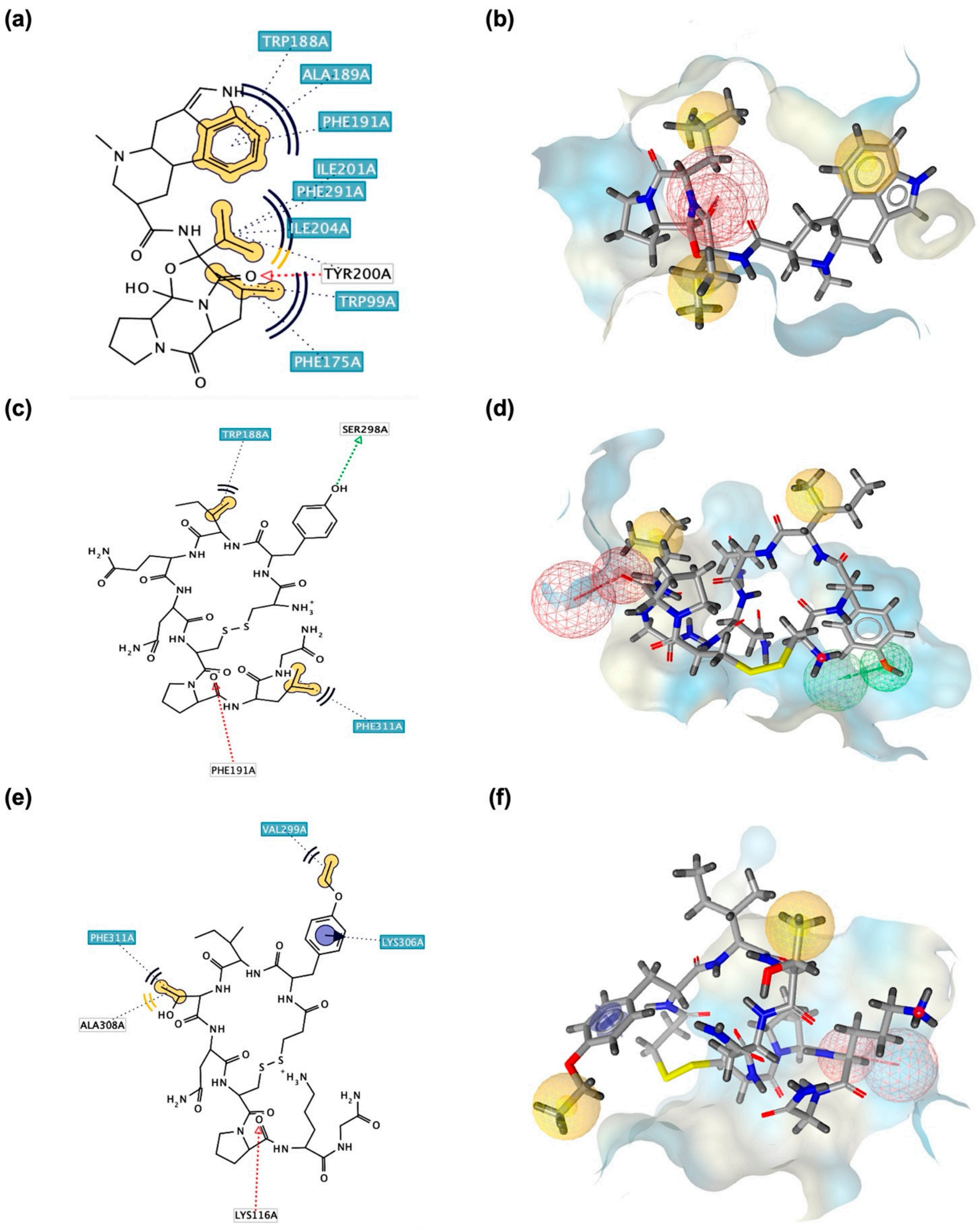

Figure S4. Pharmacophore modeling results of bromocriptine, standard agonists, and antagonists in the OXTR LBD. (**a**) 2D pharmacophore model of OXTR_Bromocriptine. (**b**) 3D pharmacophore model of OXTR_Bromocriptine. (**c**) 2D pharmacophore model of OXTR_Oxytocin (agonist). (**d**) 3D pharmacophore model of OXTR_Oxytocin (agonist). (**e**) 2D pharmacophore model of OXTR_Atosiban (antagonist). (**f**) 3D pharmacophore model of OXTR_Atosiban (antagonist).

Yellow spheres indicate hydrophobic interactions, green arrows represent hydrogen bond donors, red arrows signify hydrogen bond acceptors, and blue stars represent a positive ionizable group.
