# Supplementary material for: Molecular Insights into Bromocriptine Binding to GPCRs Within Histamine-Linked Signaling Networks: Network Pharmacology, Pharmacophore Modeling, and Molecular Dynamics Simulation
Source: Int J Mol Sci. 2025 Sep 7;26(17):8717. doi: 10.3390/ijms26178717 (PMC12428908; doi:10.3390/ijms26178717)
Supplement: Supplementary file 1 [file ijms-26-08717-s001.zip › Supplementary Data S7 - Molecular Dynamics (MD) Simulation.pdf]

Supplementary File S7

Molecular Dynamics (MD) Simulation of Top Ligand-Receptor Complexes

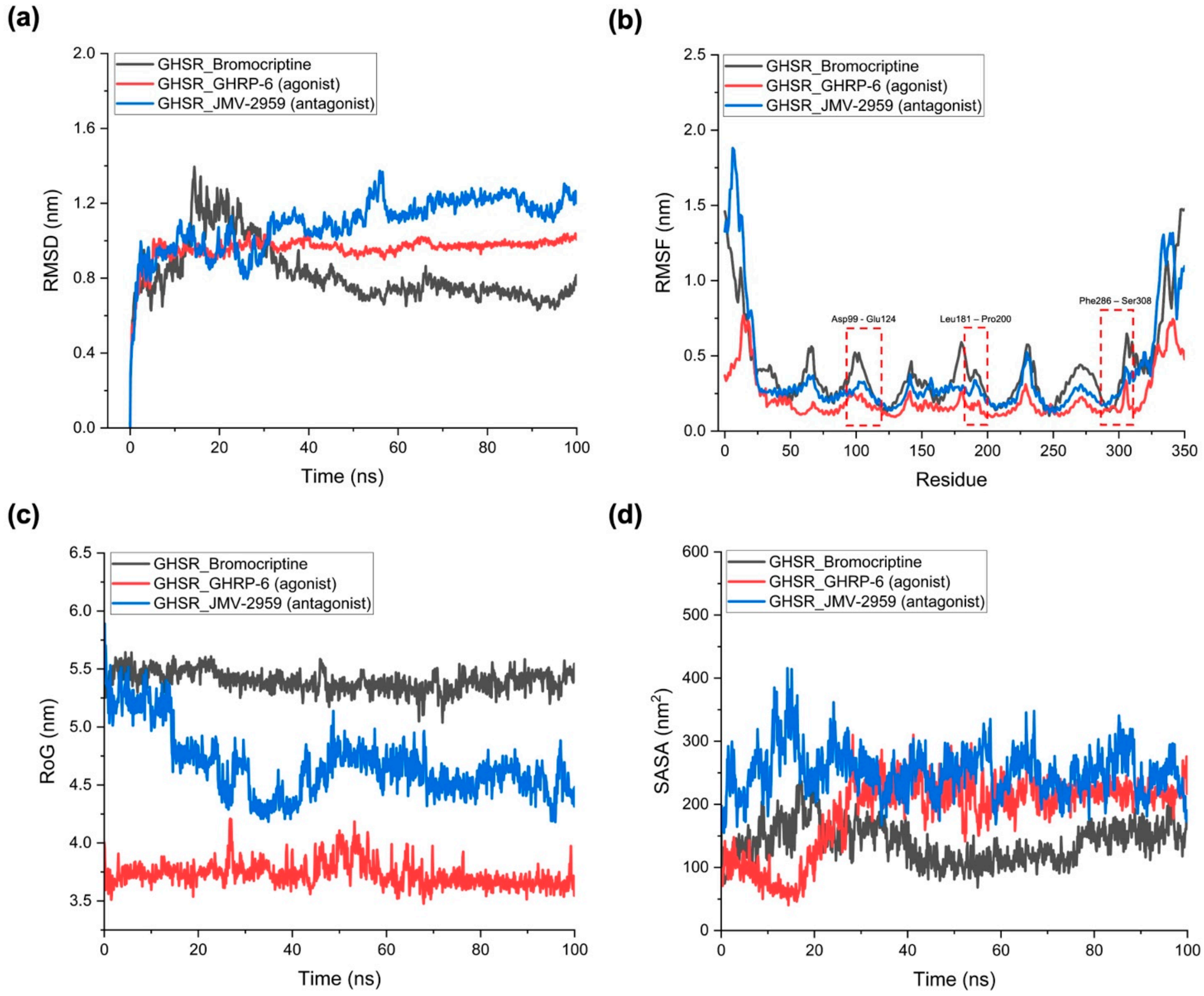

Figure S5. MD simulation profiles of bromocriptine, standard agonists, and antagonists bound to the GHSR LBD. (**a**) Root mean square deviation (RMSD). (**b**) Root mean square fluctuation (RMSF). (**c**) Radius of gyration (RoG). (**d**) Solvent-accessible surface area (SASA).

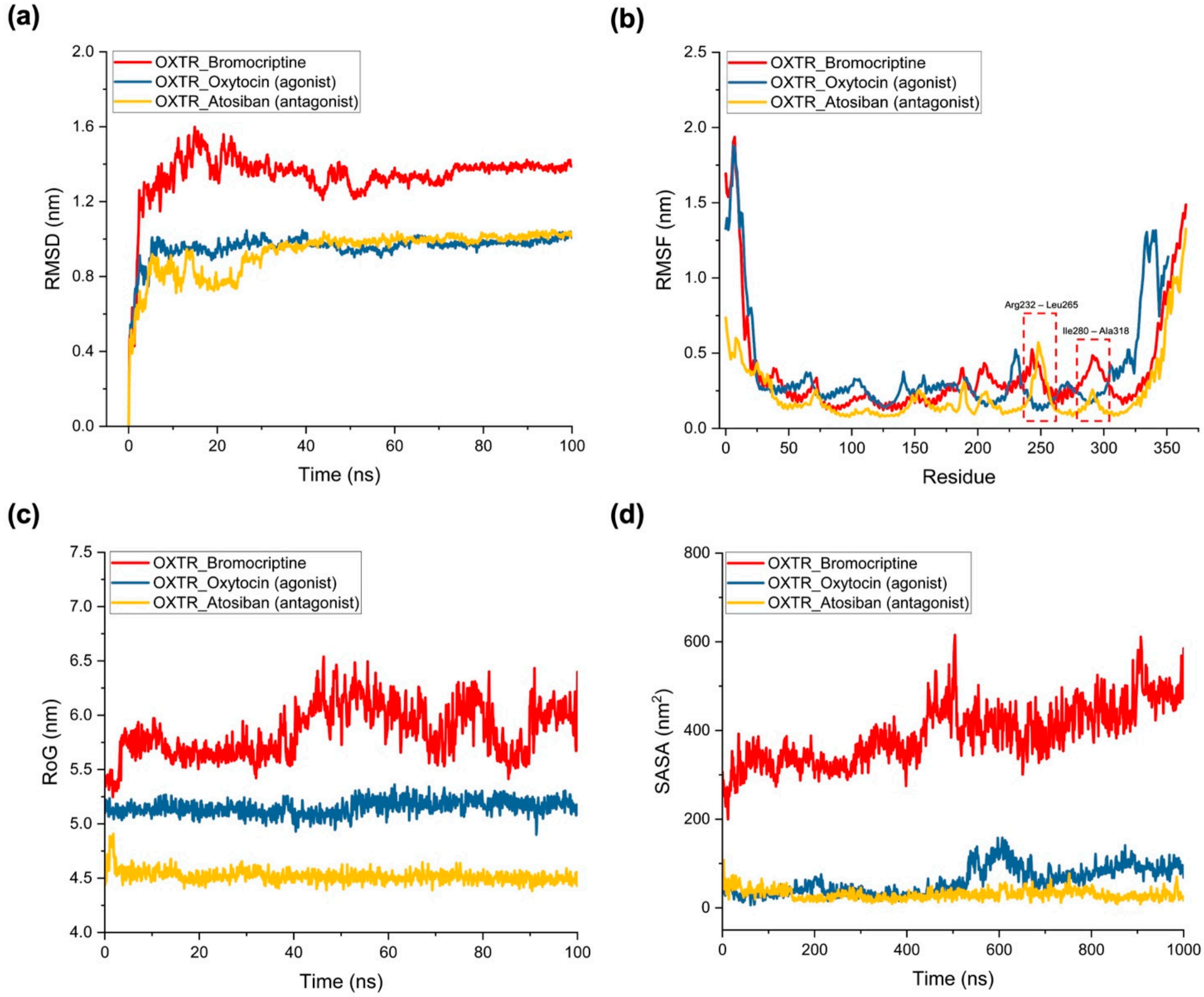

Figure S6. MD simulation profiles of bromocriptine, standard agonists, and antagonists bound to the OXTR LBD. (**a**) Root mean square deviation (RMSD). (**b**) Root mean square fluctuation (RMSF). (**c**) Radius of gyration (RoG). (**d**) Solvent-accessible surface area (SASA).
